# The laterodorsal tegmentum-ventral tegmental area circuit controls depression-like behaviors by activating ErbB4 in DA neurons

**DOI:** 10.1038/s41380-021-01137-7

**Published:** 2021-05-14

**Authors:** Hongsheng Wang, Wanpeng Cui, Wenbing Chen, Fang Liu, Zhaoqi Dong, Guanglin Xing, Bin Luo, Nannan Gao, Wen-Jun Zou, Kai Zhao, Hongsheng Zhang, Xiao Ren, Zheng Yu, Heath L. Robinson, Zhipeng Liu, Wen-Cheng Xiong, Lin Mei

**Affiliations:** 1 Department of Neurosciences, School of Medicine, Case Western Reserve University, Cleveland, OH USA; 2 Clinical and Experimental Therapeutics, University of Georgia, Augusta, GA USA; 3 Louis Stokes Cleveland Veterans Affairs Medical Center, Cleveland, OH USA

**Keywords:** Neuroscience, Depression

## Abstract

Dopamine (DA) neurons in the ventral tegmental area (VTA) are critical to coping with stress. However, molecular mechanisms regulating their activity and stress-induced depression were not well understood. We found that the receptor tyrosine kinase ErbB4 in VTA was activated in stress-susceptible mice. Deleting ErbB4 in VTA or in DA neurons, or chemical genetic inhibition of ErbB4 kinase activity in VTA suppressed the development of chronic social defeat stress (CSDS)-induced depression-like behaviors. ErbB4 activation required the expression of NRG1 in the laterodorsal tegmentum (LDTg); LDTg-specific deletion of NRG1 inhibited depression-like behaviors. NRG1 and ErbB4 suppressed potassium currents of VTA DA neurons and increased their firing activity. Finally, we showed that acute inhibition of ErbB4 after stress attenuated DA neuron hyperactivity and expression of depression-like behaviors. Together, these observations demonstrate a critical role of NRG1-ErbB4 signaling in regulating depression-like behaviors and identify an unexpected mechanism by which the LDTg-VTA circuit regulates the activity of DA neurons.

## Introduction

Depression is a common and debilitating disorder. Patients with depression suffer from symptoms including amotivation, anhedonia, and social withdrawal, suggesting the involvement of the rewarding system in pathophysiology of depression. Ventral tegmental area (VTA) dopamine (DA) neurons are critical to processing salient signals [1] and modulating reward, aversion, and stress responses [2–4]. They respond differently to appetitive and aversive stimuli (acute or chronic), and mediate different responses [5–8]. For example, in response to chronic social defeat stress (CSDS), the activity of VTA DA neurons is reduced in resilient mice because of increased K^+^ currents, whereas K^+^ currents are hardly changed in susceptible mice [9]. However, underlying regulatory mechanisms of potassium (K^+^) channels are not well understood. Nevertheless, inability of VTA DA neurons to adapt to and cope with long-term stress has been implicated in depression pathology [9–11]. VTA DA neurons are tightly regulated by neurons of different brain regions. For example, in response to stress, VTA-projecting neurons of the ventral pallidum (VP) and norepinephrine (NE) neurons in the locus coeruleus (LC) increase in firing, which are required for susceptibility and resilience of depression-like behaviors, respectively [12–14]. On the other hand, chemogenetic inhibition of cholinergic neurons in LDTg or blockade of acetylcholine receptors (AChRs) in VTA inhibits the firing of VTA DA neurons and prevents the development of depression-like behaviors. However, how these circuits regulate the activity of VTA DA neurons remains unclear.

ErbB4 is a receptor tyrosine kinase of the EGF receptor family; it binds to and thus is activated by NRG1, a trophic factor [15–18]. In the cortex, hippocampus and amygdala (AMY), ErbB4 is expressed specifically in GABAergic interneurons [19–21]. The NRG1-ErbB4 signaling has been implicated in the assembly of the GABAergic circuits of the cortex, hippocampus, and AMY during development [22–24] and the excitation-inhibition (E-I) balance by promoting GABA release from interneurons in adulthood [25–27]. Although ErbB4 is known to be expressed in DA neurons [28], its functions appear to be controversial. For example, DA levels were found increased in the cortex and striatum by exogenous NRG1, but reduced by transgenic NRG1 [29–33]. In addition, pharmacological inhibition or genetic mutation of ErbB4 was shown to increase, reduce or have no effect on DA levels in the cortex, striatum or nucleus accumbens (NAc) [31, 34, 35]. Nevertheless, both *NRG1* and *ERBB4* are among the 269 risk genes identified by genome-wide association study (GWAS) of 246,363 depression patients [36]. Genetic variants of *NRG1* and *ERBB4* appear to relate to responses to classical antidepressants [37–39]. However, whether and how abnormal NRG1-ErbB4 signaling contributes to development and/or expression of depression-like behaviors remains unclear.

Here we provide evidence that the kinase activity of ErbB4 in the VTA was increased in mice susceptible to the CSDS. Depression-like behaviors were suppressed by mutating or inhibiting ErbB4 in VTA DA neurons, revealing a necessary role of ErbB4 and its activity in depression-like behaviors. Interestingly, NRG1, the ligand to activate ErbB4, was not produced in the VTA, but in LDTg. We showed that NRG1 in the LDTg was increased by CSDS and was required for ErbB4 activation in VTA DA neurons and depression-like behaviors. In vivo recording revealed that the firing of VTA DA neurons was increased by NRG1 but inhibited by chemical genetic inhibition of ErbB4. We investigated mechanisms by which NRG1 regulates the activity of VTA DA neurons, and explored the effect of acute inhibition of ErbB4 in VTA on moue behavior. Our results demonstrate a critical role of NRG1-ErbB4 signaling in development and expression of depression-like behaviors via inhibiting K^+^ channels of VTA DA neurons. These observations identify an unexpected mechanism that in response to stress, the LDTg-VTA circuit releases NRG1 to regulate VTA DA neuron excitability and provide insight into pathological mechanisms of depression.

## Materials and methods

### Mice

DAT-IRES-Cre mice (006660), Rosa-CAG-LSL-tdTomato-WPRE mice (007905; Ai9) and ErbB4-Cre ERT2 mice (012360) were purchased from The Jackson Laboratory. Floxed ErbB4 (B4f/f) mice, T796G mice, and floxed NRG1 (NRG1f/f) mice were described previously [27, 40, 41]. Mice were housed in environment of 22 ± 2 °C and 12/12 h light/dark cycle, with free access to food and water. Animal procedures were approved by the Institutional Animal Care and Use Committees of Case Western Reserve University. Male mice of 10 to 16 weeks were used and randomly allocated to different groups. Regarding estimating sample size, we followed previous similar experiments and used sample size calculator at ClinCalc.com (http://clincalc.com/Stats/SampleSize.aspx). In behavioral tests, electrophysiological recordings and biochemical analyses, the observers were blinded to genotypes and/or treatments.

### Immunofluorescence and imaging

Mice were treated with isoflurane and transcardially perfused with phosphate buffered saline (PBS) and 4% paraformaldehyde (PFA). Brains were collected and immersed in 4% PFA for 2–4 h at room temperature (RT). Fixed brains were transferred to 20% and 30% sequentially sucrose for dehydration. Dehydrated brains were imbedded in Cryo-Embedding Compound (Cat# 16362, Ted Pella Inc., Redding, California, USA) and sectioned into 20~40-µm-thick slices with cryostat (HM550, Thermo Scientific). Slices were washed with PBS (30 min), permeabilized with PBST (0.5% Triton X-100 in PBS, 30 min) and blocked with 10% donkey serum (in PBST, 1 h, RT). Incubation of primary antibody (in 10% donkey serum) was performed overnight at 4 °C. After washing with PBS (10 min × 3 times), slices were incubated in secondary antibody (2 h, RT). After wash with PBS, slices were mounted onto slides and sealed with SlowFade® Diamond Antifade Mountant (cat. 36972, ThermoFisher). Antibodies used included: chicken anti-GFP (1:2000, Cat# GFP-1020, AVES, RRID:AB_10000240); rabbit anti-TH (1:1000, Cat# MAB318, Chemicon, RRID:AB_2201528). Images were captured with Zeiss LSM 800 confocal microscope. AAV expression at LDTg and dorsal raphe nucleus (DRN) was analyzed by the Keyence BZ-X800 system.

### Dissection of brain regions

Dissection of VTA, NAc, LDTg, DRN, VP, AMY, and medial prefrontal cortex (mPFC) were performed in freshly prepared brain slices under stereo microscope. After exposure to isoflurane, mice were transcardially perfused with ice-cold PBS. Brains were collected and sectioned into 200-μm-thick slices [42] in ice-cold oxygenated section solution (see components for in vitro electrophysiology). Slices containing the target nuclei were transferred to agarose-coated Petri dish, and the target nuclei were collected by punches under stereo microscope. During the operation, the slices were incubated in oxygenated section solution. Dissected tissues from different slices for the same nucleus were pooled and mRNA and proteins were extracted. Locations of the targeted nuclei were referred to the mouse brain atlas prepared by Paxinos and Franklin [43].

### Western blot

Brain tissues were homogenized in RIPA lysis buffer (50 mM Tris-HCl, pH 7.4, 150 mM NaCl, 2 mM EDTA) containing 0.5% sodium deoxycholate, 0.1% SDS, 1 mM PMSF, 1 mM Na_3_VO_4_, 1 mM NaF, 1 mM DTT and protease inhibitor cocktail (ThermoFisher, 11697498001). Samples were resolved on SDS-PAGE and transferred to the nitrocellulose membranes (Cat# 1620112, Bio-Rad). After blocking with 5% BSA (bovine serum albumin) in PBS-T, PBS containing 0.5% Tween-20), membranes were incubated in primary antibody (in 5% BSA) overnight at 4 °C. Membranes were washed with PBS-T (10 min × 3) and incubated in HRP-conjugated secondary antibody (Life Technology) at RT for 1 h. After wash, the immunoreactive bands were visualized by applying enhanced chemiluminescence substrates (Cat# 32106, Pierce), signals were captured with LI-COR Odyssey infrared imaging system. Antibodies used included: rabbit anti-ErbB4 (1:2000, 0618, generously provided by Cary Lai); rabbit anti-phospho-ErbB4 (1:200, Cat# 4757, Cell Signaling Technology, RRID:AB_2099987); mouse anti-β-actin (1:5000, Cat# A1978, Sigma, RRID:AB_476692); mouse anti-phospho-ERK (1:500, Cat# 9106, Cell Signaling Technology, RRID:AB_331768); rabbit anti-ERK (1:500, Cat# 9102, Cell Signaling Technology, RRID:AB_330744); mouse anti-NRG1 (1:200, Cat# MA5-12896, Invitrogen, RRID:AB_10986946).

### Preparation and injection of afatinib, lapatinib and 1NMPP1 to VTA

Two milligram of afatinib (Cat# S1011, Selleckchem, USA) or lapatinib (Cat# S2111, Selleckchem, USA) was first dissolved in 40-μl DMSO to get solution A. On vortexer, solution A was diluted by gradually adding solvent B (2% DMSO + 5% Tween 80 in water) to a volume of 500 μl to get solution C. Addition of solvent B should be slow to avoid formation of pellet and the solution C obtained should be clear. A large volume of PBS was added to solution C to realize the work solution, at concentration of 10 nM and 100 nM for afatinib and lapatinib, respectively. In the work solution, concentrations of DMSO and Tween were about 0.00001% (v/v). One milligram of 1NMPP1 (Cat# 529581, Millipore Sigma) was dissolved in 60-μl DMSO on vortexer to get stock solution (50 mM). Before use, the stock solution was diluted with PBS to a concentration of 100 nM.

For single injection of drugs into VTA, guide cannulae (26 gauge, Plastics 1) were implanted into brain, with the tips of cannulae at 0.5 mm above the VTA. The guide cannulae were affixed on the skull with dental cement and dummy cannulae were placed in the guide cannulae to avoid contamination. After surgery, mice were allowed to recover for one week. At the day of drug injection, internal cannulae (extend beyond the guide cannula for 0.5 mm) (33 gauge, Plastics 1) were inserted into the guide cannulae and drugs were infused (30 nl/min) into target brain regions driven by a microPump (UMP3, World Precision Instruments).

For consecutive injection of 1NMPP1 into VTA, osmotic pumps (ALZET^®^ Osmotic Pumps) were used. The pump was implanted subcutaneously at the nape and connected to a cannula (Plastics One, 33 gauge) that was inserted into VTA and affixed on the skull with dental cement. Physiochemical property of the osmotic materials in the pump and the osmotic pressure from the tissue environment displace the prefilled 1NMPP1 solution from the pump at a constant and predetermined rate (110 nl/h).

### Microinjection of AAV viruses and drugs

Microinjection was performed with glass pipette (Cat# 5-000-1001-X10, Drummond) pulled with a micropipette puller (P1000, Sutter, USA). The outer diameter of the tip of the pipette was 30–50 μm. The tips of glass pipettes were polished (Micro Forge MF-830, Narishige, Japan). Glass pipettes were equipped on a 2.5-μl Hamilton syringe and connected to a microPump (UMP3, World Precision Instruments). Injection rate was set at 20–30 nl/min. After infusion, the pipette was left at the injection site for 5 min. For local deletion or overexpression of ErbB4 or NRG1 gene, AAV-Cre-GFP, AAV-GFP (UNC Vector Core), or AAV-NRG1-GFP (generated by the lab, see below) was injected to the VTA (200 nl, AP: −3.0 mm, ML: ± 0.58 mm, DV: −4.45 mm) or LDTg (300 nl, AP: −4.50 mm, ML: ± 0.50 mm, DV: −3.55 mm). Injection of recombinant NRG1 (rHRG β177-244) [44] and 1NMPP1 (Cat# 529581, Millipore Sigma) into VTA followed the same protocol.

### Generation of AAV-NRG1-GFP

NRG1Iβ1a (from Dr. Douglas Falls, Emory University) was cloned into the MCS region of pAAV-IRES-hrGFP. An HA tag was inserted between the Ig domain and EGF domain. Generation of the AAV expressing NRG1 followed a protocol described previously [45]. Plasmids of pAAV-NRG1Iβ1a, helper plasmid (pHelper) and pUCmini-Icap-PHP were co-transfected into HEK293 cells. HEK293 cells were cultured in DMEM containing 5% (vol/vol) FBS, 1 × non-essential amino acids, and 50 U/ml Penicillin–streptomycin. Five days after transfection, the AAV were harvested and purified with iodixanol gradient ultracentrifugation. Titration was performed with the qPCR method and AAV products of 10^12^ vg/ml were used for the following studies.

### In vivo electrophysiology

Single unit recording of DA neurons was performed as previously described with minor modifications [46]. Briefly, extracellular glass electrodes were pulled from glass tubing (1B150F, World Precision Instruments, FL). The electrodes were filled with 2 M NaCl and 2% biocytin with resistance set to ~15 MΩ. Putative DA neurons were identified according to criteria reported [47]: (1) firing rate < 12 Hz, (2) triphasic spike width more than 2 ms, and (3) burst firing pattern. Burst firing occurs when two consecutive spikes show an inter-spike interval < 80 ms; burst firing terminated when no spike was observed within 160 ms afterwards [48]. Data were submitted to spike sorting using etos3.0 [49]; single-unit clusters were identified with Klusters and NeuroScope [50]. Analyses of unit data was conducted using home-made scripts for Matlab. After recording, neurons were labeled by biocytin after application of positive currents; the colocalizaiton of biocytin and TH signals after immunostaining indicated the recorded neurons as DA neurons.

### In vitro electrophysiology

Ex vivo electrophysiology was performed as previously described [9, 51]. Coronal slices of 250-µm thickness were prepared in ice-cold oxygenated sectioning buffer (in mM, 120 Choline Chloride, 2.5 KCl, 0.5 CaCl_2_, 7 MgSO_4_, 1.25 NaH_2_PO_4_, 5 Sodium ascorbate, 3 Sodium pyruvate, 26 NaHCO_3_, and 25 glucose) with vibratome (Leica, 1200S). Slices were incubated in oxygenated sectioning buffer at 34 °C for 15 min and recovered in oxygenated artificial cerebrospinal fluid (ACSF) (in mM, 124 NaCl, 2.5 KCl, 2.5 CaCl_2_, 2 MgCl_2_, 1.25 NaH_2_PO_4_, 26 NaHCO_3_, and 10 glucose) at RT for at least 1 h. Slices were incubated with oxygenated ACSF (2 ml/min, 32 ± 1 °C) during recording.

Cells were visualized with upright microscope equipped with 40 × water immersion lens (BX51WI, Olympus) and infrared-sensitive CCD camera (C2400-75, Hamamatsu). ErbB4 positive neurons were labeled by tdTomato by crossing the ErbB4-CreER mice with the Ai9 reporter mice. Recorded neurons were labeled with biocytin and co-staining with anti-TH revealed the DAergic identity. tdTomato+ neurons were recorded in whole-cell patch-clamp configuration with pipettes (3–5 MΩ) filled with internal solution (in mM, 125 K-gluconate, 5 KCl, 10 HEPES, 0.2 EGTA, 1 MgCl_2_, 4 Mg-ATP, 0.3 Na-GTP, 10 phosphocreatine, 2.7 biocytin, pH 7.25, 290–300 mOsm). Action potentials (AP) were recorded in DA neurons that were clamped at −70 mV and injected with incremental currents of 25-pA step from 0 to 200 pA [9]. Series resistance was monitored throughout the experiments and cells of series resistance > 20 MΩ were discarded. Data were collected by Multiclamp 700B amplifier and 1550B digitizer (Molecular Devices). Data were sampled at 10 kHz and filtered at 3 kHz.

To determine effects by synaptic inputs on VTA DA neurons, tdTomato+ neurons were voltage-clamped at −70 mV. miniature EPSCs (mEPSCs) and spontaneous EPSCs (sEPSCs) were recorded with pipettes (input resistance: 3–5 MΩ) filled with internal solution (in mM, 125 K-gluconate, 5 KCl, 10 HEPES, 0.2 EGTA, 1 MgCl_2_, 4 Mg-ATP, 0.3 Na-GTP, 10 phosphocreatine, pH 7.25, 290–300 mOsm) in the presence of 100 µM picrotoxin (PTX) to block GABA_A_ receptors. 1 μM TTX was added for mEPSC recording. Spontaneous IPSCs (sIPSCs) and miniature IPSCs (mIPSCs) were recorded with pipettes (input resistance: 2–4 MΩ) filled with internal solution (in mM, 130 CsCl, 10 HEPES, 0.2 EGTA, 1 MgCl_2_, 4 Mg-ATP, 0.3 Na-GTP, 10 phosphocreatine and 5 QX-314, pH 7.25, 290–300 mOsm), in the presence of DL-AP5 (100 μM) and CNQX (20 μM) to block glutamate receptors-mediated currents. 1 μM TTX was added for mIPSC recording.

Voltage-gated K^+^ channel-mediated currents in DA neurons, held at −70 mV, were measured with increasing voltages −70 to +20 mV (at 10-mV step, 4 s) in the presence of 1 µM TTX, 200 µM CdCl_2_, 1 mM kynurenic acid, and 100 µM PTX as described previously [9]. A-type K^+^ currents (*I*_*A*_) and delayed rectifier K^+^ currents (*I*_*DR*_) were recorded as previously described with modifications [52, 53]. *I*_*A*_ was isolated by adding 20 mM TEA (Tetraethylammonium) in the perfusion buffer to block *I*_*DR*_. To characterize the steady-state activation of *I*_*A*_, outward K^+^ currents were measured in DA neurons, held at −100 mV, with increasing voltages from −80 to +40 mV (at 15-mV step, 200 ms). To characterize *I*_*A*_ inactivation, neurons were held at −90 mV and stimulated with a 10-s prepulse (stepping from −120 to −40 mV in 10 mV increments) and recorded for outward K^+^ currents for 200 ms after the membrane potentials were switched to −20 mV. To study the steady-state activation of *I*_*DR*_, neurons were held at −70 mV and stimulated with a 500-ms prepulse of −40 mV, and recorded for outward K^+^ currents for 200 ms after the membrane potentials were switched to test voltages (stepping from −80 to +40 mV in 15 mV increments). Activation or inactivation values were fitted with single Boltzmann function with Graphpad to reveal V_h_ (voltage for half-maximal activation or inactivation) and k (slope factor of curves).

### Behavioral tests

CSDS and subthreshold social defeat (subSD) were performed as previously reported [5, 7]. In CSDS, tested mice received 10 consecutive days of social defeat. Each day, the tested mouse was introduced into the home cage of an unfamiliar CD1 aggressor and received a 5-min physical attack. After physical attack, the tested mouse was maintained in the cage of aggressor, which was partitioned into two halves with a transparent and perforated plexiglass plate. The CD1 aggressors were prescreened and those showed an attack latency < 30 s in three tests were employed. Severe tissue damage was avoided. After social defeat procedure, tested mice were individually housed until all tests finished. The paradigm of subSD consisted of two rounds of 2-min physical attack and 10-min sensory contact within one day, intermitted by a 5-min rest period in the home cage of the tested mice.

Social avoidance and sucrose preference were tested 24 h post the final social defeat. Social avoidance was tested in a plastic box (50 cm × 50 cm × 20 cm), with an overhead camera monitoring the behaviors. The test included two 2.5-min sessions. In the first session, a mesh cup was located in the middle of one side of the box, tested mouse was allowed to freely explore the whole box. A 8-cm wide corridor surrounding the mesh cup was defined as interaction zone (IZ). Tested mice were put back to the home cage and an unfamiliar CD-1 male mouse was introduced into the mesh cup. After that, the tested mouse was put into the test box again and another 2.5-min activity was recorded. Tracking software (EthoVision; Noldus) was used to analyze the locomotion and the time spent in IZ. Interaction ratio (IR) was calculated by (time in IZ with presence of social target)/(time in IZ with absence of social target) × 100; after CSDS, mice with IR < 100 were grouped as susceptible, while other mice were grouped as resilient.

Sucrose preference test was performed at the home cage of the tested mice which were individually housed. Two bottles containing water and 2% sucrose, respectively were placed on the wire lid. Every 12 h, the two bottles were weighted and their positions were switched. Sucrose preference = (Consumed sucrose)/(Consumed sucrose + Consumed water) × 100%.

### Statistical analysis

GraphPad Prism was used for data analysis. Data normality was determined by the D’Agostino-Pearson normality test. Difference between two groups was analyzed by unpaired Student’s *t* test; difference among three or more groups was analyzed by one-way ANOVA followed by Sidak’s or Tukey’s multiple comparisons test; difference in current injection-induced spikes or total outward K^+^ currents was analyzed by two-way ANOVA followed by Sidak’s multiple comparisons test (see Figure legends for details). Results for the spontaneous firing, current injection-induced spikes, and K^+^ currents were expressed as mean ± SEM; other data were expressed as box-and-whisker plots. *P* value < 0.05 for two-sided tests was considered significant.

## Results

### ErbB4 in VTA DA neurons is necessary for depression-like behaviors

**Fig. 1:**
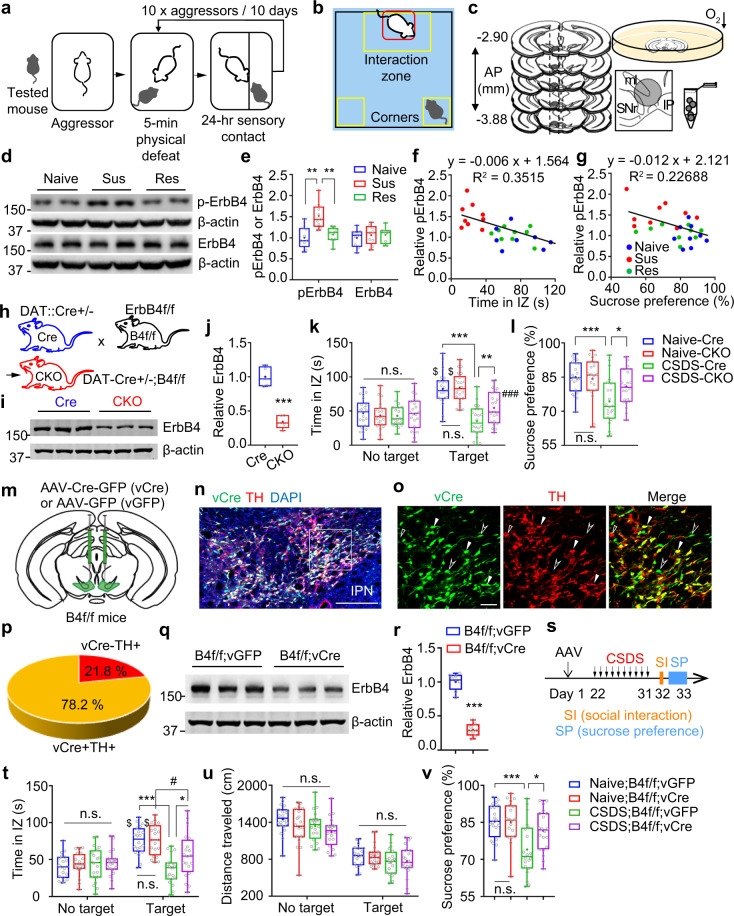
Requirement of ErbB4 in VTA DA neurons for the development of depression-like behaviors. **a** Procedure of chronic social defeat stress (CSDS), with CD1 breeders as aggressors. **b** Diagram of social avoidance test. **c** Diagram showing micro-dissection of VTA. Dissection was performed in oxygenated cutting solutions under a stereo microscope; oval or round shape of gray color indicating the regions of interest in brain slices, which were punched and pooled. **d, e** Increased pErbB4 in VTA of CSDS-susceptible mice. Sus susceptible, Res resilient. **d** Representative western blots. **e** Quantitative data. Data were expressed as box-and-whisker plots, error bars indicated the min and max data points, center lines indicated the median, plus symbols indicated the mean, circles were values for all individual samples. For pErbB4: One-way ANOVA, *F*_(2, 15)_ = 8.326, **P = 0.0037, Tukey’s multiple comparisons test, **P_(Naive vs. Sus)_ = 0.0049, *P*_(Naive vs. Res)_ = 0.85018, *P_(Sus vs. Res)_ = 0.0146; *n* = 9 mice per group. **f, g** Negative correlation between VTA pErbB4 level and social interaction time **f** and sucrose preference value **g**; IZ time spent in social interaction zone; correlation between pErbB4 and IZ, **P = 0.0011; correlation between pErbB4 and sucrose preference value, *P = 0.0120; *n* = 9 for naive, Sus, and Res group, respectively. **h–j** Conditional knockout (CKO) of ErbB4 in DA neurons. **h** DAT-Cre (Cre) mice crossed with the ErB4f/f (B4f/f) mice to generate the DAT-Cre;B4f/f (CKO) mice; **i** Western blot showing reduced ErbB4 protein in VTA in CKO mice; **j** quantitative data of **i**, Data were expressed as box-and-whisker plots. Unpaired Student’s *t* test, *t*_8_ = 8.704, ***P < 0.001; *n* = 5 mice per group. **k** Attenuation of CSDS-induced social avoidance in CKO mice. Notice that mice heterozygous in DAT-Cre gene were used. IZ, time spent in interaction zone. Data were expressed as box-and-whisker plots. Unpaired Student’s *t* test; Target vs. No Target in Naive-Cre mice, *t*_42_ = 5.232, ^$^*P* < 0.0001; Target vs. No Target in Naive-CKO mice, *t*_42_ = 6.69, ^$^*P* < 0.0001; With presence of Target: *t*_(42)(Naive-Cre vs. CSDS-Cre)_ = 6.764, ***P < 0.0001; *t*_(42) (Naive-CKO vs. CSDS-CKO)_ = 4.356, ^# # #^*P* < 0.0001; *t*_(42)(CSDS-Cre vs. CSDS-CKO)_ = 2.762, **P = 0.0085; *n* = 22 mice per group. n.s. no statistical difference. **l** Attenuation of CSDS-induced reduction of sucrose preference in CKO mice. Data were expressed as box-and-whisker plots. Unpaired Student’s *t* test, *t*_(42)(Naive-Cre vs. CSDS-Cre)_ = 4.094 ***P = 0.0002; *t*_(42)(CSDS-Cre vs. CSDS-CKO)_ = 2.378, *P = 0.0221; *n* = 22 mice per group. **m**–**r** VTA-specific deletion of ErbB4 by expressing AAV-Cre (vCre) in VTA of B4f/f mice. **m** Anatomic diagram showing injection of vCre or AAV-GFP (vGFP) into bilateral VTAs. **n** Representative image showing expression of vCre at VTA, scale bar, 250 µm. **o** Enlargement of rectangle area in **n**, scale bar, 50 µm. **p** Quantification of DA neurons (TH+) that express vCre. **q** Representative western blots for dissected VTA showing decreased ErbB4 protein in vCre-injected B4f/f mice (B4f/f;vCre), compared with vGFP-injected mice (B4f/f;vGFP). **r** Quantification of **q**. Data were expressed as box-and-whisker plots. Unpaired Student’s *t* test, *t*_14_ = 12.89, ***P < 0.001; *n* = 8 mice per group. **s** Time scheme for behavioral studies. **t** Attenuation of CSDS-induced social avoidance in B4f/f;vCre mice. IZ, time spent in interaction zone. Data were expressed as box-and-whisker plots. Unpaired Student’s *t* test; Target vs. No Target in Naive;B4f/f;vGFP mice, *t*_38_ = 6.363, ^$^*P* < 0.0001, Target vs. No Target in Naive;B4f/f;vCre mice, *t*_38_ = 5.755, ^$^*P* < 0.0001. With presence of Target: *t*_(38)(Naive;B4f/f;vGFP vs. CSDS;B4f/f;vGFP)_ = 6.598, ***P < 0.0001; *t*_(38)(Naive-B4f/f;vCre vs. CSDS;B4f/f;vCre)_ = 2.592, ^#^*P* = 0.0135; *t*_(38)(CSDS;B4f/f;vGFP vs. CSDS;B4f/f;vCre)_ = 2.43, *P = 0.0199. *n* = 20 mice per group. **u** No alteration in locomotion of B4f/f;vCre mice. **v** Attenuation in CSDS-induced reduction of sucrose preference in B4f/f;vCre mice. Data were expressed as box-and-whisker plots. Unpaired Student’s *t* test, *t*_(38)(Naive;B4f/f;vGFP vs. CSDS;B4f/f;vGFP)_ = 3.965, ***P = 0.0003; *t*_(38)(CSDS;B4f/f;vGFP vs. CSDS;B4f/f;vCre)_ = 2.491, *P = 0.0172. *n* = 20 mice per group. (Also see Supplementary Fig. S1 for the segregation of susceptible and resilient mice; Supplementary Fig. S2 for the verification of DAT-Cre mice; Supplementary Fig. S3 for the unaltered expression TH in VTA; Supplementary Fig. S4 for the tracking images of social avoidance; Supplementary Fig. S5 for the unaltered locomotion of CKO mice; Supplementary Fig. S6 for the AAV expression at VTA.).

To investigate the role of ErbB4 in depression-like behaviors, we determined whether its level or activity is changed in the VTA under stress. Mice were exposed to CSDS for 10 days [54] and subjected to social avoidance test (Fig. 1a, b) and sucrose preference test, two well established paradigms for CSDS-induced depressive-like symptoms [7], on day 11 and 12, respectively. Mice were scored for interaction ratio with a CD1 social target and sucrose preference, and were segregated into susceptible and resilient groups (Supplementary Fig. S1). On day 13, the VTA was dissected from freshly prepared brain slices (Fig. 1c) and probed for ErbB4 protein and its phosphorylation by western blotting. As shown in Fig. 1d, e, ErbB4 level was similar among susceptible, resilient and naive mice (without CSDS), suggesting CSDS had little effect on ErbB4 protein level. Interestingly, ErbB4 tyrosine phosphorylation (pErbB4) was increased in susceptible mice, compared with that of naive or resilient mice, indicative of increased ErbB4 activity in the VTA in CSDS-susceptible mice. Furthermore, pErbB4 level in VTA was negatively correlated with both social interaction and sucrose preference (Fig. 1f, g). These results suggest a potential involvement of ErbB4 signaling in depression-like symptoms. ErbB4 in the VTA is expressed in DA neurons [19, 28, 33, 55–58]. To investigate the contribution of ErbB4 to CSDS-induced depression-like behaviors, we deleted the ErbB4 gene from DA neurons by crossing ErbB4f/f mice (referred to as B4f/f) with DAT-Cre mice that express Cre specifically in DA neurons starting from E15 [59]. When DAT-Cre mice were crossed with Ai9 reporter mice (Supplementary Fig. S2a), tdTomato was expressed in neurons that were stained positive for tyrosine hydroxylase (TH), a marker of the DA neurons (Supplementary Fig. S2b). 92% of VTA neurons were double positive for tdTomato and TH (Supplementary Fig. S2c). Because the insertion of the Cre gene reduces the expression of DAT in DAT-Cre mice [59], only DAT-Cre+/−; B4f/f (referred to as CKO) and DAT-Cre+/− (referred to as Cre, as control) mice were studied and mice homozygous for Cre were excluded. As shown in Fig. 1h–j, ErbB4 protein level was reduced in the VTA of CKO mice, compared with Cre mice. The residual weak band of ErbB4 in CKO samples might come from non-DA neurons or tissues. Western blot analysis showed similar TH levels in VTA between the two genotypes (Supplementary Fig. S3), suggesting proper DA neuron differentiation.

Naive Cre and CKO mice (referred to as Naive-Cre and Naive-CKO, respectively) spent more time in the interaction zone in the presence of a social target (Fig. 1b, k), and there was no difference between the genotypes (Fig. 1k, Supplementary Fig. S4a), suggesting normal social interaction in CKO mice. After CSDS, the two genotypes spent similar time in the interaction zone in the absence of a social target (Fig. 1k), suggesting that basal exploration behavior was not altered by CKO. When a social target was present, CSDS-Cre and CSDS-CKO mice spent less time in the interaction zone, compared with their naive states (Fig. 1k). However, CSDS-CKO mice spent more time in the interaction zone, compared with CSDS-Cre mice (Fig. 1k, Supplementary Fig. S4a), indicating a necessary role of ErbB4 for CSDS-induced depression-like behavior. Note that locomotion activity was similar between the two genotypes with or without a social target (Supplementary Fig. S5). Likewise, sucrose preference was similar between Naive-Cre and Naive-CKO mice (Fig. 1l), suggesting ErbB4 deletion had little effect on basal sucrose preference. Compared with naive mice, CSDS reduced sucrose preference in Cre mice (Fig. 1l). Interestingly, the preference of CSDS-CKO mice was more than that of CSDS-Cre mice (Fig. 1l), in support of reduced depression-like symptoms by ErbB4 CKO. Together these observations identify a necessary role of ErbB4 in DA neurons in regulating CSDS-induced depression-like behaviors.

Cre in DAT-Cre mice is expressed in DA neurons in the entire brain [60]. To determine that ErbB4 in VTA neurons is critical, AAV-CMV-Cre-EGFP or AAV-CMV-EGFP (referred to as vCre and vGFP, respectively) was injected into bilateral VTAs of B4f/f mice (referred to as B4f/f;vCre and B4f/f;vGFP, respectively) (Fig. 1m and Supplementary Fig. S6). To validate AAV infection of DA neurons, sections were examined for expression of GFP and TH (Fig. 1n, o), which showed that 78% of TH+ cells expressed GFP (Fig. 1p). ErbB4 protein was reduced in VTA of B4f/f;vCre, compared with B4f/f;vGFP mice (Fig. 1q, r). The time of Naive-B4f/f;vGFP and Naive-B4f/f;vCre spent in the interaction zone was increased at similar levels by a social target (Fig. 1t), in agreement with data of CKO mice. After CSDS and when a social target was absent, time spent in the interaction zone was similar between B4f/f;vCre and B4f/f;vGFP mice (Fig. 1t). Both groups of mice spent less time in interaction zone when a social target was present, compared with their naive states (Fig. 1t). However, the time in interaction zone was increased in CSDS-B4f/f;vCre mice, compared with CSDS-B4f/f;vGFP mice (Fig. 1t). As observed with CKO mice, VTA-specific reduction of ErbB4 had little effect on locomotor activity (Fig. 1u). These results suggest that ErbB4 in the VTA is necessary for CSDS-induced depression-like behavior. This notion was supported by increased sucrose preference of CSDS-B4f/f;vCre, compared with CSDS-B4f/f;vGFP mice (Fig. 1v). Together with studies of CKO mice, a parsimonious interpretation of the results is that ErbB4 in VTA DA neurons is necessary for the development of depression-like behaviors.

### Reduced depression-like behaviors after inhibiting ErbB4 kinase activity in VTA

**Fig. 2:**
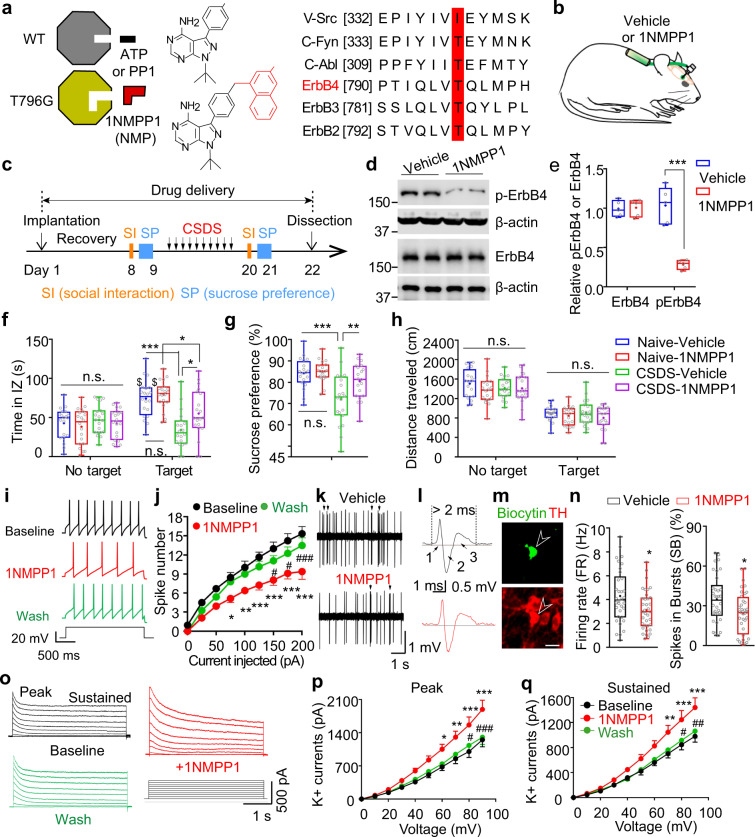
Attenuated development of depression-like behaviors after consecutive chemical genetic inhibition of ErbB4 kinase activity in VTA. **a** Generation of the T796G mice by mutating the threonine 796 to glycine; the mutation causes the enlargement of the ATP-binding pocket which could be accessed by the bulk competitive inhibitor, 1NMPP1. **b** Diagram showing continuous infusion of 1NMPP1 or Vehicle into VTA via an implanted osmotic pump. **c** Time scheme for the experiments. **d**, **e** Inhibition of pErbB4 by consecutive injection of 1NMPP1 into VTA. **d** Representative western blots showing reduced pErbB4 in VTA of 1NMPP1-treated mice; **e** Quantitative data of **d**; Data were expressed as box-and-whisker plots. Unpaired Student’s *t* test; for pErbB4, *t*_8_ = 5.533, ***P = 0.0006; *n* = 5 mice per group. **f** Attenuation of CSDS-induced social avoidance in 1NMPP1-treated mice. IZ time spent in interaction zone. Data were expressed as box-and-whisker plots. Unpaired Student’s *t* test; Target vs. No Target in Vehicle mice, *t*_38_ = 4.519, ^$^*P* < 0.0001; Target vs. No Target in 1NMPP1 mice, *t*_38_ = 6.382, ^$^*P* < 0.0001; With presence of Target: *t*_(38)(Naive-Vehicle vs. CSDS-Vehicle)_ = 5.331, ***P < 0.0001; *t*_(38)(Naive-1NMPP1 vs. CSDS-1NMPP1)_ = 3.086, ^# #^*P* = 0.0038; *t*_(38)(CSDS-Vehicle vs. CSDS-1NMPP1)_ = 2.417, *P = 0.0206; *n* = 20 mice per group. **g** Attenuated development of CSDS-induced reduction in sucrose preference in 1NMPP1-treated mice. Data were expressed as box-and-whisker plots. Unpaired Student’s *t* test, *t*_(38)(Naive-Vehicle vs. CSDS-Vehicle)_ = 5.331, ***P < 0.0001; *t*_(38)(Naive-1NMPP1 vs. CSDS-1NMPP1_) = 3.086, ^# #^*P* = 0.0038; *t*_(38)(CSDS-Vehicle vs. CSDS-1NMPP1)_ = 2.417, *P = 0.0206; *n* = 20 mice per group_._ **h** No alteration in locomotion after 1NMPP1 treatment. **i**–**q** Reduced firing of VTA DA neurons by inhibition of ErbB4 activity, via enhancing K^+^ currents. **i**–**j** Recording in VTA slices from DAT-Cre;Ai9;T796G mice showing decreased current injection-induced spikes in DA neurons by incubation with 1NMPP1. **i** Representative current injection-induced spikes. **j** Quantification of **i**. Data were expressed as mean ± SEM. Two-way ANOVA, Baseline vs. 1NMPP1, *F*_(interaction)(8, 304)_ = 2.03, *P = 0.0427; Sidak’s multiple comparisons test, *P_(75 pA)_ = 0.0220, **P_(100 pA)_ = 0.0074, ***P_(125 pA)_ = 0.0005, ***P_(150 pA)_ < 0.0001, ***P_(175 pA)_ < 0.0001, ***P_(200 pA)_ < 0.0001; 1NMPP1 vs. Wash, *F*_(interaction)(8, 304)_ = 1.374, *P* = 0.2070; *F*_(main effect of 1NMPP1)(1,38)_ = 65.22, ^# # #^*P* < 0.0001; Sidak’s multiple comparisons test, ^#^*P*_(150pA)_ = 0.0327, ^#^*P*_(175 pA)_ = 0.0242, ^# # #^*P*_(200pA)_ = 0.0009; *n* = 20 cells from 8 mice. **k**–**n** In vivo recordings performed in T796G mice showing decreased firing rate (FR) and percentage of spikes in bursts (SB) after 1NMPP1 injection into VTA. **k** Representative firing traces of VTA DA neurons shortly (20 min) after injection of Vehicle or 1NMPP1 into VTA. Arrowheads indicated some of the spikes that fired in bursts. **l** Representative APs of DA neurons. Arrowheads and Arabic numerals indicated the three phases. **m** Representative immunostaining images showing recorded neuron double positive of biocytin and TH. Scale bar, 20 µm. **n** Quantitative data in **m**, data were expressed as box-and-whisker plots. Unpaired Student’s *t* test: *t*_(FR)(68)_ = 2.558 *P = 0.0128; *t*_(SB)(68)_ = 2.532, *P = 0.0137; *n* = 34 (Vehicle) or 36 (1NMPP1) cells from 13 mice. **o**–**q** In vitro recording in VTA slices from T796G mice showing enhancement of total K^+^ currents by 1NMPP1. **o** Representative traces of total K^+^ currents in DA neurons after 1NMPP1 incubation. **p–q** Quantitative data in **o** showing enhancement of both the peak and sustained phases by 1NMPP1. Data were expressed as mean ± SEM. Two-way ANOVA. For peak phase: Baseline vs. 1NMPP1, *F*_(interaction)(9, 414)_ = 3.099, **P = 0.0013, Sidak’s multiple comparisons test, ***P_(90 mV)_ < 0.0001, ***P_(80 mV)_ = 0.0001, **P_(70 mV)_ = 0.0042, *P_(60 mV)_ = 0.0377; 1NMPP1 vs. Wash, *F*_(interaction)(9, 414)_ = 2.099, ^#^*P* = 0.0285, Sidak’s multiple comparisons test, ^# # #^*P*_(90 mV)_ = 0.0001, ^#^*P*_(80 mV)_ = 0.0115. For sustained phase: Baseline vs. 1NMPP1, *F*_(interaction)(9, 414)_ = 2.723, **P = 0.0043, Sidak’s multiple comparisons test, ***P_(90 mV)_ < 0.0001, ***P_(80 mV)_ = 0.0007, **P_(70 mV)_ = 0.0064; 1NMPP1 vs. Wash, *F*_(interaction)(9, 414)_ = 1.779, *P* = 0.0704; *F*_(main effect of 1NMPP1)(1, 46)_ = 23.37, ^# # #^*P* < 0.0001, Sidak’s multiple comparisons test, ^# #^*P*_(90 mV)_ = 0.0025, ^#^*P*_(80 mV)_ = 0.0131. *n* = 24 cells of 10 mice for each group. (Also see Supplementary Fig. S7 for the verification of positions of cannulae; Supplementary Fig. S10 for inhibition of BDNF release at NAc by 1NMPP1 injected into VTA of CSDS-exposed mice.).

ErbB4 is a receptor tyrosine kinase, and its kinase activity is required for acute GABA release from interneurons [27, 61, 62]. To determine whether ErbB4 kinase activity is required for CSDS-induced depression-like behaviors, we investigated the behavioral changes after ErbB4 inhibition in T796G mice. T796G is a strain of chemical genetic ErbB4 mutant where by mutating threonine 796 (T796) to glycine (G), its ATP-binding pocket is enlarged and thus accessible by the bulky inhibitor 1NMPP1 (Fig. 2a) [27]. T796G mice develop normally without apparent deficits in neurotransmission and behaviors; however, treatment with 1NMPP1 suppresses GABA release and impairs top-down attention in free-moving mice [27]. To inhibit ErbB4 activity, 1NMPP1 was delivered to the VTA by a cannula connected to an osmotic pump implanted beneath the skin in the nape (Fig. 2b). The positions of the tip of the cannulae were verified by postmortem histological examination (Supplementary Fig. S7). 1NMPP1 delivery during CSDS reduced pErbB4, but not ErbB4 protein, compared with mice infused with vehicle (referred to as Veh) (Fig. 2d, e). As shown in Fig. 2f, g, 1NMPP1 had little effect on social interaction and sucrose preference of naive T796G mice; however, it increased social interaction and sucrose preference of CSDS-stressed mice. As a control, 1NMPP1 had no effect on the time spent in interaction zone without social target (Fig. 2f) or total traveled distance (Fig. 2h). These results suggest VTA ErbB4 kinase activity contributes to the development of depression-like behaviors.

### Reduced firing of VTA DA neurons by ErbB4 inhibition

To investigate how ErbB4 regulates DA neurons, VTA slices were isolated from T796G;DAT-Cre;Ai9 mice and DA neurons (tdTomato+) were recorded. Interestingly, 1NMPP1 decreased the number of APs in response to injected currents (Fig. 2i, j), indicating that ErbB4 activity is necessary for maintaining the excitability of DA neurons. To determine whether this mechanism occurs in vivo, glass-pipettes were implanted into the VTA of T796G mice for extracellular, single-unit recording. DA neurons in VTA generated characteristic APs [triple phases, long duration (>2 ms), and slow firing rate (<12 Hz)] (Fig. 2k, l), some of which appeared in bursts (Fig. 2k) [47, 63]. Neurons with this firing pattern were confirmed to be TH+ by biocytin which was delivered into recorded neurons through electrodes under continuous electrophysiological monitoring (Fig. 2m). As shown in Fig. 2n, 1NMPP1 injection into VTA decreased both the firing rate and the number of bursts, compared with Veh-injected mice. These results indicate a necessary role of ErbB4 activity in maintaining the activity of VTA DA neurons in vivo. To investigate the mechanisms underlying the changes in DA neuron excitability, we characterized sEPSCs, mEPSCs, sIPSCs and mIPSCs in DA neurons and as shown in Supplementary Fig. S8, they were similar before and after 1NMPP1 treatment, suggesting that synaptic inputs onto VTA DA neurons were not altered by ErbB4 kinase. 1NMPP1 had no effect on intrinsic properties of DA neurons such as resting membrane potential, input resistance and membrane capacitance (Supplementary Table S1). However, 1NMPP1 increased the amplitude of after-hyperpolarization (AHP) and elongated first spike latency upon current injection (Supplementary Table S1), revealing a potential mechanism of how ErbB4 regulates DA neuron excitability.

To investigate how ErbB4 regulates DA neuron activity, we characterized the effects of 1NMPP1 on K^+^ currents (Fig. 2o–q). Changes in the activity of K^+^ channels have been implicated in VTA DA neurons after CSDS exposure [7, 64]; however, the upstream regulatory mechanisms remained unclear. K+ currents were recorded at different voltages in tdTomato+ neurons at various clamping voltages. The recording was conducted in the presence of TTX and CdCl2 to block Na+ and Ca2+ currents [9]. 1NMPP1 increased the amplitudes of K^+^ currents at the peak and sustained phases. The effect of 1NMPP1 was reversible as it was diminished by medium change (Fig. 2o–q). These results indicated that K^+^ currents in VTA DA neurons was enhanced by 1NMPP1 and suggested that ErbB4 kinase activity maintains DA neuron activity by suppressing K^+^ channel activity. Further analysis of transient (*I*_*A*_) and sustained (*I*_*DR*_) components of K^+^ currents indicated that 1NMPP1 caused left-ward shift of *I*_*A*_ activation curve, with little effect on *I*_*A*_ inactivation curve or *I*_*DR*_ activation curve (Supplementary Fig. S9). These results suggest that enhanced *I*_*A*_ activation may be a mechanism of reduced DA neuron activity by 1NMPP1. Increased activity of VTA DA neurons could increase BDNF release from their terminals in the NAc, which is necessary for depression-like symptoms in response to social stress [54, 65, 66]. As previously reported, CSDS increased the BDNF in NAc (Supplementary Fig. S10c, d). Interestingly, BDNF levels in NAc were reduced by 1NMPP1 (100 nM) in the CSDS-susceptible mice (Supplementary Fig. S10c, d). However, 1NMPP1 had no effect on mRNA levels of BDNF in NAc (Supplementary Fig. S10e), in agreement with previous reports that BDNF is released from VTA-originated axon terminals [67–70]. Altogether, these results suggest that ErbB4 signaling in VTA is necessary for the firing of VTA DA neurons and the stress-induced BDNF release in NAc.

### CSDS-induced expression of NRG1 in LDTg neurons for depression-like behaviors

**Fig. 3:**
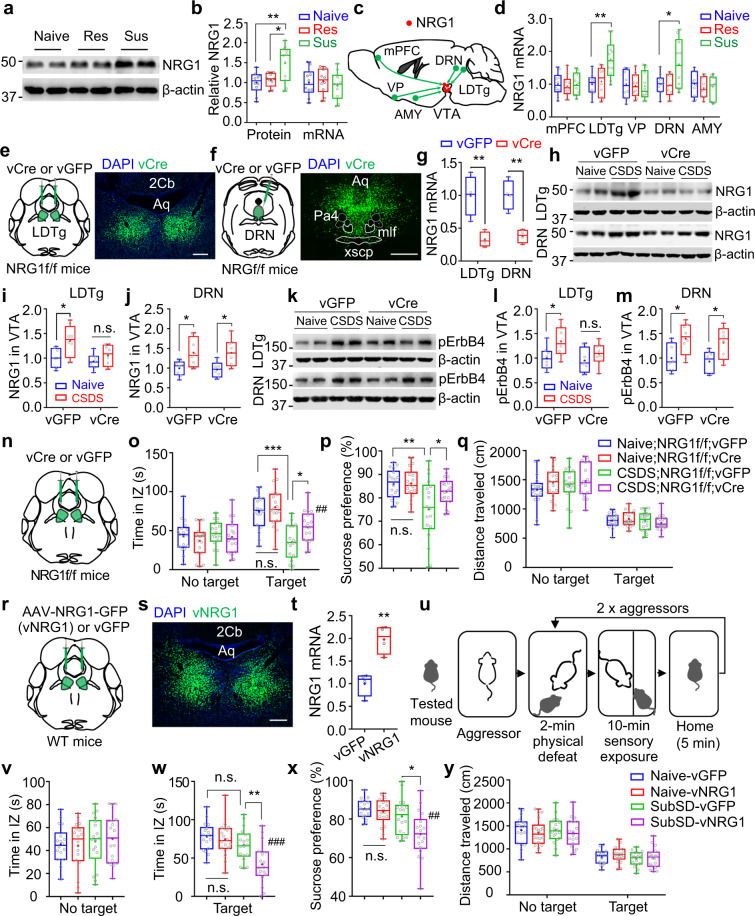
Increased expression of NRG1 in LDTg after CSDS and its requirement for the development of depression-like behaviors. **a**, **b** Increased NRG1 protein, but not mRNA, in VTA of CSDS-susceptible mice. **a** Representative western blots for dissected VTA; **b** Quantitative data of NRG1 protein and mRNA in VTA. Sus susceptible, Res resilient. Data were expressed as box-and-whisker plots. For western blot, one-way ANOVA, *F*_(2, 12)_ = 6.785, **P = 0.0041; Sidak’s multiple comparisons test, **P_(Naive vs. Sus)_ = 0.0065, *P_(Res vs. Sus)_ = 0.0249; *n* = 10 (Naive), 9 (Res), or 11 (Sus) mice. **c** Diagram showing brain regions that send excitatory afferents to VTA DA neurons and are involved in social defeat-induced behavioral changes. mPFC medial prefrontal cortex, VP ventral pallidum, AMY amygdala, DRN dorsal raphe nucleus, VTA ventral tegmental area, LDTg laterodorsal tegmentum. **d** Increased NRG1 mRNA in LDTg and DRN, not mPFC, VP, or AMY in Sus mice. Data were expressed as box-and-whisker plots. One-way ANOVA. For LDTg, *F*_(2, 27)_ = 8.185, **P = 0.0017; Tukey’s multiple comparisons test, **P_(Naive vs. Sus)_ = 0.0028, **P_(Res vs. Sus)_ = 0.0076. For DRN, *F*_(2, 27)_ = 5.125, *P = 0.0130; Tukey’s multiple comparisons test, *P_(Naive vs. Sus)_ = 0.0302, *P_(Res vs. Sus)_ = 0.0227. *n* = 10 mice per group. **e**–**j** Deletion of NRG1 gene in LDTg reduced NRG1 protein in VTA in CSDS-exposed mice. **e**–**g** vCre injection into LDTg and DRN of NRG1f/f mice reduced local NRG1 mRNA. **e**, **f** vCre expression in LDTg and DRN; Left panels anatomic diagrams, right panels representative images of vCre expression, 2Cb 2nd cerebellar lobule, Aq aqueduct, Pa4 paratrochlear nucleus, mlf medial longitudinal fasciculus, xscp decussation of the superior cerebellar peduncle. Scale bars, 500 µm. **g** Reduced mRNA in LDTg and DRN detected by qRT-PCR. Data were expressed as box-and-whisker plots. Unpaired Student’s *t* test: *t*_(6)(LDTg)_ = 4.112, **P = 0.0063; *t*_(6)(DRN)_ = 5.056, **P = 0.0023; *n* = 4 mice per group. **h**–**j** Reduced NRG1 protein in VTA after injecting vCre into LDTg, but not DRN. Mice after indicated paradigms were analyzed. **h** Representative western blots for dissected VTA; **i, j** Quantitative data in **h**. Data were expressed as box-and-whisker plots. Unpaired Student’s *t* test; for LDTg, *t*_(14)(Naive-vGFP vs. CSDS-vGFP)_ = 2.688, *P = 0.0177; *t*_(14)(Naive-vCre vs. CSDS-vCre)_ = 1.352, *P* = 0.1979, *n* = 8 mice per group; for DRN, *t*_(14)(Naive-vGFP vs. CSDS-vGFP)_ = 2.725, *P = 0.0164;, *t*_(14)(Naive-vCre vs. CSDS-vCre)_ = 3.046, **P = 0.0087, *n* = 8 mice per group. **k**–**m** Reduced pErbB4 in VTA after vCre injection into LDTg, but not DRN. Mice after indicated paradigms were analyzed. **k** Representative western blots for dissected VTA; **l, m** Quantitative data in **k**. Data were expressed as box-and-whisker plots. Unpaired Student’s *t* test; for LDTg, *t*_(14)(Naive-vGFP vs. CSDS-vGFP)_ = 2.554, *P = 0.0230; *t*_(14)(Naive-vCre vs. CSDS-vCre)_ = 1.18, *P* = 0.2577, *n* = 8 mice per group; for DRN, *t*_(14)(Naive-vGFP vs. CSDS-vGFP)_ = 2.629, *P = 0.0198; *t*_(14)(Naive-vCre vs. CSDS-vCre)_ = 2.845, *P = 0.0130, *n* = 8 mice per group. **n**–**y** Requirement of NRG1 in LDTg for the development of depression-like behaviors. **n**–**q** Deletion of NRG1 in LDTg attenuated the development of social avoidance and reduction in sucrose preference. **n** Anatomical diagram showing injection of vCre or vGFP into LDTg of NRG1f/f mice. **o** Attenuation of CSDS-induced social avoidance in NRG1f/f;vCre mice. Data were expressed as box-and-whisker plots. Unpaired Student’s *t* test, *t*_(34)(Naive-vGFP vs. CSDS-vGFP)_ = 5.371, ***P < 0.0001; *t*_(34)(Naive-vCre vs. CSDS-vCre)_ = 3.409, ^# #^*P* = 0.0017; *t*_(34)(CSDS-vGFP vs. CSDS-vCre)_ = 2.718, *P = 0.0103; *n* = 18 mice per group. **p** Attenuated development of CSDS-induced reduction of sucrose preference in NRG1f/f;vCre mice. Data were expressed as box-and-whisker plots. Unpaired Student’s *t* test; *t*_(34)(Naive-vGFP vs. CSDS-vGFP)_ = 3.08, **P = 0.0041; *t*_(34)(CSDS-vGFP vs. CSDS-vCre)_ = 2.2, *P = 0.0347; *n* = 18 mice per group. **q** No change in locomotion in NRG1f/f;vCre mice. **r**–**t** Injection of AAV-NRG1-GFP (vNRG1) into LDTg increased local expression of NRG1. **r** Anatomic diagram showing injection of vNRG1 or vGFP into LDTg of WT mice; **s** Representative image showing expression of vNRG1 at LDTg. Aq aqueduct, 2Cb 2nd cerebellar lobule. Scale bar, 1 mm. **t** Increased NRG1 mRNA detected by qRT-PCR. Data were expressed as box-and-whisker plots. Unpaired Student’s *t* test, *t*_6_ = 4.865, **P = 0.0028. *n* = 4 mice per group. **u**–**y** Enhanced development of depression-like behaviors by overexpressing NRG1 in LDTg after exposure to subSD. **u** Diagram showing the procedure of subSD. **v** No alteration in time spent in social target-absent IZ. **w** Reduction in time spent in social target-present IZ after subSD in vNRG1-injected mice. Data were expressed as box-and-whisker plots. Unpaired Student’s *t* test; *t*_(34)(Naive-vGFP vs. subSD-vGFP)_ = 1.686, *P* = 0.1009; *t*_(34)(Naive-vNRG1 vs. subSD-vNRG1)_ = 4.088, ^# # #^*P* = 0.0003; *t*_(34)(subSD-vGFP vs. subSD-vNRG1)_ = 3.237, **P = 0.0027; *n* = 18 mice per group. **x** Reduction in sucrose preference after subSD in vNRG1-injected mice. Data were expressed as box-and-whisker plots. Unpaired Student’s *t* test, *t*_(34)(Naive-vGFP vs. subSD-vGFP)_ = 1.565, *P* = 0.1269; *t*_(34)(Naive-vNRG1 vs. subSD-vNRG1)_ = 3.553, ^# #^*P* = 0.0011; *t*_(34)(subSD-vGFP vs. subSD-vNRG1)_ = 2.591, *P = 0.0140; *n* = 18 mice per group. **y** No changes in locomotion in vNRG1-injected mice. (Also see Supplementary Fig. S11 for AAV expression at DRN and LDTg; Supplementary Fig. S12 for the unaltered expression of total ErbB4 in VTA; Supplementary Fig. S12 for increased NRG1 in VTA after subSD in vNRG1-injected mice.).

Neuronal activity has been shown to increase NRG1 expression by increasing transcription and activity-dependent release [71–73]. CSDS induction of pErbB4 in VTA suggests an increase in NRG1. Indeed, western blot analysis showed an increase in NRG1 protein in CSDS-susceptible mice, compared with naive and resilient mice (Fig. 3a, b). However, this increase was not associated with an elevation in NRG1 mRNA in the VTA (Fig. 3b), suggesting that NRG1 may be delivered to VTA from upstream neurons. VTA DA neurons receive inputs from mPFC, VP, AMY, DRN, and LDTg (Fig. 3c), where the activity of projection neurons regulates social stress-induced depression-like behaviors [12, 74–79]. To identify neurons in which regions are critical, we analyzed NRG1 mRNA in these regions. As shown in Fig. 3d, NRG1 mRNA in mPFC, VP, or AMY was similar among naive, susceptible and resilient groups. In contrast, NRG1 mRNA was increased in DRN and LDTg of susceptible mice, compared with naive and resilient mice (Fig. 3d). To verify their contribution to increased NRG1 in VTA, we ablated NRG1 expression in LDTg and DRN by injecting vCre virus into LDTg (Fig. 3e, Supplementary Fig. S11a, b) and DRN (Fig. 3f, Supplementary Fig. S11c, d) of NRG1f/f mice. Three weeks after virus injection, NRG1 mRNA in LDTg and DRN was reduced (Fig. 3g). Remarkably, CSDS-induced increase in NRG1 protein in VTA was diminished by vCre injection in LDTg, compared with vGFP-injected mice (Fig. 3h, i). In accord, CSDS-induced pErbB4 in VTA was reduced by vCre injection into LDTg (Fig. 3k, l). vCre injection into to DRN seemed to have little effect on CSDS-induced increase in NRG1 protein (Fig. 3h, j) and pErbB4 (Fig. 3k, m) in VTA. Note that viral injection had little effect on total level of ErbB4 in VTA (Supplementary Fig. S12a–d). These results suggest that LDTg is required for CSDS-increased NRG1 protein and activation of ErbB4 in VTA.

Next, we determined whether LDTg-originated NRG1 is required for CSDS-induced depression-like behaviors by injecting NRG1f/f mice with vCre or vGFP into LDTg (referred to as NRG1f/f;vCre and NRG1f/f;vGFP mice, respectively) (Fig. 3n). Three weeks after viral injection, NRG1f/f;vCre and NRG1f/f;vGFP mice showed no difference in the time spent in social target-absent interaction zone before or after CSDS (Fig. 3o). CSDS;NGR1f/f;vGFP mice spent less time in the social target-present interaction zone and consumed less sucrose; however, this effect was diminished in CSDS;NRG1f/f;vCre mice (Fig. 3o, p and Supplementary Fig. S4b). As control, the viral injections had no effect on locomotion (Fig. 3q). These results indicate that LDTg-derived NRG1 is necessary for CSDS-induced depression-like behaviors. Next, we studied the impact of increasing NRG1 level in LDTg on CSDS-induced depression-like behaviors by injecting AAV-CMV-NRG1-GFP (vNRG1) into LDTg of wild type (WT) mice (Fig. 3r, s). Three weeks after viral injection, qPCR confirmed the increased expression of NRG1 (Fig. 3t). Mice were subjected to subSD paradigm as described previously (Fig. 3u) [5, 7]. Mice after subSD do not develop depression-like behaviors but are more susceptible to subsequent stress challenges [5, 7]. In agreement, mice injected with vGFP spent a similar amount of time in the social target-present interaction zone and consumed a similar amount of sucrose after subSD, compared to the naive mice (Fig. 3v–x). In contrast, vNRG1-injected mice spent less time in social interaction and consumed less sucrose after subSD, compared with the vNRG1-injected naive mice and vGFP-injected subSD mice (Fig. 3w, x, Supplementary Fig. S4c), suggesting that NRG1 overexpression in LDTg promotes the development of depression-like behaviors. After behavior tests, we analyzed NRG1 expression in VTA by western blot, which showed an increase in vNRG1-injected, subSD-treated mice (Supplementary Fig. S12e, f). Together, these results indicate that NRG1 in LDTg is necessary and sufficient to induce depression-like behaviors after social defeat stress and suggest that the LDTg-VTA circuit may regulate VTA DA neuron activity by releasing NRG1.

**Fig. 4:**
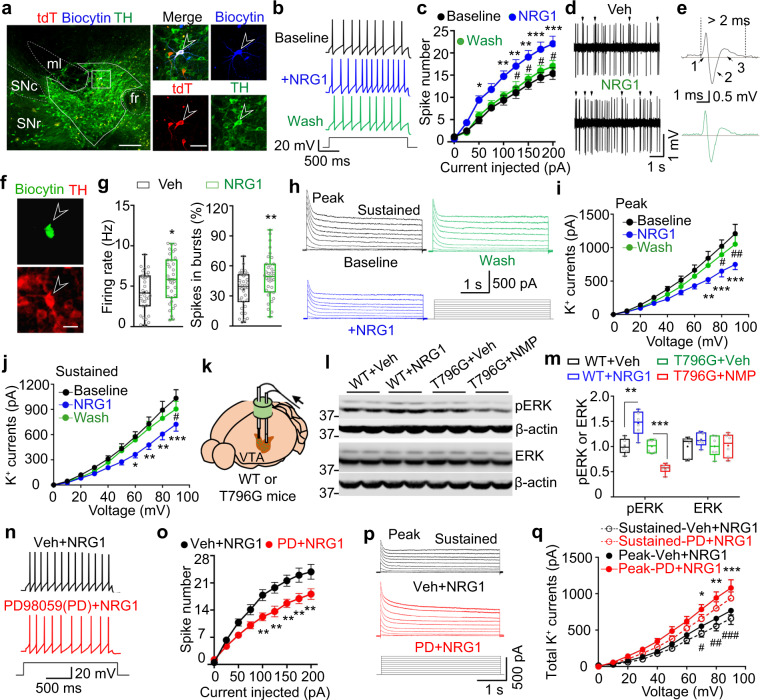
VTA DA neuron firing increased by NRG1, via suppressing K^+^ currents. **a**–**c** In vitro recording performed in VTA slices from ErbB4-CreER;Ai9 mice showing increased VTA DA neuron firing after incubation with NRG1. **a** Representative images showing recorded neurons triple positive for ErbB4 (positive of tdTomato, referred to as tdT+), TH, and biocytin. Left, VTA slices with anatomic regions. SNc substantia nigra pars compacta, SNr substantia nigra pars reticulata, ml medial lemniscus, fr fasciculus retroflexus. Right, enlargement of the rectangle area in the left image. Arrowheads indicated the tdT, TH, and biocytin triple positive neurons. Scale bars, 200 μm in the left, 50 μm in the right. To avoid overexposure of fluorescent signal, low dose of tamoxifen was used to induced a small number of tdT+ cells. **b** Representative current injection-induced spikes. **c** Quantitative data in **b**. Data were expressed as mean ± SEM. Two-way ANOVA. Baseline vs. NRG1: *F*_(interaction)(8, 288)_ = 2.081, *P = 0.0376; Sidak’s multiple comparisons test, *P_(50 pA)_ = 0.0416, **P_(100 pA)_ = 0.0085, **P_(125 pA)_ = 0.0020, **P_(150 pA)_ = 0.0012, ***P_(175 pA)_ = 0.0008, ***P_(200 pA)_ = 0.0003; NRG1 vs. Wash, *F*_(interaction)(8, 288)_ = 1.0198, *P* = 0.3001; *F*_(main effect of NRG1)(1,36)_ = 63.9, ^# # #^*P* < 0.0001; Sidak’s multiple comparisons test, ^#^*P*_(125 pA)_ = 0.0206, ^#^*P*_(150 pA)_ = 0.0228, ^#^*P*_(175 pA)_ = 0.0279, ^#^*P*_(200 pA)_ = 0.0185; *n* = 19 cells from 8 mice. **d**–**g** In vivo recordings performed in WT mice showing increased firing rate (FR) and percentage of spikes in bursts (SB) after NRG1 injection into VTA. **d** Representative firing traces of VTA DA neurons shortly (20 min) after injection of Vehicle, or NRG1 into VTA. Arrowheads indicated some of the spikes that fired in bursts. **e** Representative APs of DA neurons. Arrowheads and Arabic numerals indicated the three phases. **f** Representative immunostaining image showing recorded neuron double positive of biocytin and TH. Scale bar, 20 µm. **g** Quantitative data in **d**, data were expressed as box-and-whisker plots. Unpaired Student’s *t* test: *t*_(FR)(70)_ = 2.584, *P = 0.0119; *t*_(SB)(70)_ = 2.861, **P = 0.0056; *n* = 36 cells from 12 (Vehicle) or 13 (NRG1) mice. **h**–**j** In vitro recordings performed in VTA slices from ErbB4-CreER;Ai9 mice showing inhibition of total K^+^ currents by NRG1. **h** Representative traces of total K^+^ currents in VTA DA neurons after NRG1 incubation. **i**, **j** Quantitative data showing inhibition of both the peak and sustained phases by NRG1. Data were expressed as mean ± SEM. Two-way ANOAVA. For the peak phase: Baseline vs. NRG1, *F*_(interaction)(9, 414)_ = 3.403, ***P = 0.0005; Sidak’s multiple comparisons test, ***P_(90 mV)_ < 0.0001, ***P_(80 mV)_ = 0.0002, **P_(70 mV)_ = 0.0021; NRG1 vs. Wash, *F*_(interaction)(9, 414)_ = 1.851, *P* = 0.0577, *F*_(main effect of NRG1)(1, 46)_ = 17.97, ^# # #^*P* = 0.0001, Sidak’s multiple comparisons test, ^# #^*P*_(90 mV)_ = 0.0022, ^#^*P*_(80 mV)_ = 0.0260. For sustained phase: Baseline vs. NRG1, *F*_(interaction)(9, 414)_ = 2.402, *P = 0.0116; Sidak’s multiple comparisons test, ***P_(90 mV)_ = 0.0003, **P_(80 mV)_ = 0.0012, **P_(70 mV)_ = 0.0074; *P_(60 mV)_ = 0.0431; NRG1 vs. Wash, *F*_(interaction)(9, 414)_ = 1.449, *P* = 0.1649, *F*_(main effect of NRG1)(1, 46)_ = 23.37, ^# # #^*P* < 0.0001, Sidak’s multiple comparisons test, ^#^*P*_(70 mV)_ = 0.0417. *n* = 24 cells of 10 mice for each group. **k**–**m** pERK increased by NRG1, but decreased by NMP, injected into VTA. **k** Diagram showing acute injection of Veh, NRG1 or NMP into VTA. **l** Representative western blots for dissected VTA. **m** Quantification of l. Data were expressed as box-and-whisker plots. Unpaired Student’s *t* test, *t*_(Veh vs. NRG1)(10)_ = 3.962, **P = 0.0027; *t*_(Veh vs. 1NMPP1)(10)_ = 6.696, ***P < 0.001. **n**–**q** ERK inhibitor, PD98059 (referred to as PD), reduced the effects of NRG1 on DA neuron firing and K^+^ currents. **n** Representative traces of current injection-induced spikes in DA neurons after incubation with Veh+NRG1 or PD + NRG1. **o** Quantification of n showing reduced spikes in PD + NRG1-treated slices. Data were expressed as mean ± SEM. Two-way ANOVA, *F*_(interaction)(8, 296)_ = 2.058, *P = 0.0398; Sidak’s multiple comparisons test, **P_(100 pA)_ = 0.0087, **P_(125 pA)_ = 0.0028, **P_(150 pA)_ = 0.0054, **P_(175 pA)_ = 0.0032, **P_(200 pA)_ = 0.0063; *n* = 20 (Veh) or 19 (PD) cells from 8 mice. **p** Representative traces of total K^+^ currents after incubation with Veh+NRG1 or PD + NRG1. **q** Quantification of p showing increased K^+^ currents in PD + NRG1-treated slices. Data were expressed as mean ± SEM. Two-way ANOVA. For peak phase: *F*_(interaction)(9, 396)_ = 2.292, *P = 0.0162; Sidak’s multiple comparisons test, ***P_(90 mV)_ = 0.0002, **P_(80 mV)_ = 0.0011, *P_(70 mV)_ = 0.0154. For sustained phase: *F*_(interaction)(9, 396)_ = 2.195, ^#^*P* = 0.0216; Sidak’s multiple comparisons test, ^# # #^*P*_(90 mV)_ = 0.0003, ^# #^*P*_(80 mV)_ = 0.0022, ^#^*P*_(70 mV)_ = 0.0207; *n* = 22 cells of 8 mice for each group. (Also see Supplementary Fig. S13 for quantification of ErbB4-expressing DA neurons in VTA; Supplementary Fig. S10 for the increased BDNF release at NAc after injection of NRG1 into VTA in subSD-exposed mice.).

Our hypothesis predicts that NRG1, released from LDTg terminals, would regulate DA neuron activity. To test this, we recorded ErbB4+ neurons in VTA slices of ErbB4-CreER;Ai9 mice where ErbB4+ neurons were visualized by tdTomato [19]. Recorded neurons were back labeled by biocytin and stained with anti-TH antibody (Fig. 4a–c). We focused on neurons that were double positive for tdTomato and TH (Fig. 4a and Supplementary Fig. S13). As shown in Fig. 4b, c, NRG1 increased the number of APs in response to injected currents, suggesting increased activity of DA neurons. In addition, in WT mice, local injection of NRG1 increased both the firing rate and percentage of spikes in bursts, compared with Veh-treated group (Fig. 4d–g), indicative of increased VTA DA neuron activity by NRG1 in vivo. However, NRG1 had little effect on synaptic inputs onto VTA DA neurons (Supplementary Fig. S8) but inhibited the AHP and decreased the first spike latency (Supplementary Table S1). These results support the hypothesis that NRG1 regulates depression-like behaviors by increasing the firing of VTA DA neurons. In support of this notion, NRG1 was able to suppress K^+^ currents (such as *I*_*A*_ activation) in VTA DA neurons (Fig. 4h–j; Supplementary Fig. S14). In addition, injection of NRG1 into VTA increased BDNF in NAc of subSD-exposed mice (Fig. S10c, d).

NRG1 activation of ErbB4 activates the ERK pathway [15, 18]. ERK signaling in VTA has been shown to be required for DA neuron firing and behavioral sensitization to CSDS [80, 81]. Next, we investigated potential participation of the ERK pathway. As shown in Fig. 4k–m, NRG1 increased whereas 1NMPP1 reduced phosphorylation of ERK (pERK) in the VTA. Importantly, PD98059, an inhibitor of ERK activation, attenuated the effects of NRG1 on current injection-induced spikes (Fig. 4n, o) and K^+^ currents (Fig. 4p, q) of DA neurons. Together, these findings reveal that the NRG1-ErbB4 signaling of the LDTg-VTA circuit is critical to the activity of DA neurons via ERK-dependent inhibition of K^+^ currents.

### Amelioration of depression-like behaviors by acute inhibition of VTA ErbB4

The above results demonstrate a necessary role of NRG1 and ErbB4 in CSDS-induced depression-like behaviors. To determine whether ErbB4 kinase activity is required for the expression of depression-like behaviors, we first injected afatinib and lapatinib, inhibitors of ErbB kinases, into bilateral VTAs (Supplementary Fig. S15a). Afatinib has IC50 values of 0.5 nM for ErbB1, 1 nM for ErbB4, and 14 nM for ErbB2 whereas lapatinib is more specific for ErbB1 and ErbB2 (IC50 values being 9.2 and 10.8 nM, respectively), but not ErbB4 (IC50 of 367 nM) [82–84]. In accord, pErbB4 was reduced in the VTA by afatinib (10 nM, 200 nl), but not by lapatinib (100 nM, 200 nl), and the inhibitory effect of afatinib occurred within 30 min, persisted 24 h, but recovered 48 h, after injection (Fig. S15b, c). To determine their effects on behavior expression, WT mice were subjected to CSDS, and susceptible mice were injected with the two inhibitors 30 min before social avoidance test, followed by sucrose preference test (Supplementary Fig. S15d). As shown in Supplementary Fig. S15e, without a social target, the time spent in the interaction zone by different groups of mice was similar. In contrast, compared with Veh- or lapatinib-treated mice, time in interaction zone with social target was increased in afatinib-injected mice, 30 min and 24 h, but not 48 h, after injection (Supplementary Fig. S15f). Note that afatinib or lapatinib had little effect on locomotor behavior (Supplementary Fig. S15g, h). These results suggest a necessary role of VTA ErbB4 kinase activity in expression of CSDS-induced social avoidance. Moreover, afatinib-injected mice increased sucrose preference 24 h after injection, compared with Veh- or lapatinib-injected mice; and again, this effect disappeared 48 h after injection (Supplementary Fig. S15i). We also compared the social avoidance and sucrose preference before and after afatinib injection, results of which indicate that both parameters are improved (Supplementary Fig. S15f, i). Considering the key role of increased DA neuron activity in the depression-like behaviors, we recorded the current injection-induced firing of DA neurons after incubation with afatinib or lapatinib (Supplementary Fig. S15j–l). The results showed attenuation of DA neuron hyperactivity after incubation with afatinib, but not lapatinib (Supplementary Fig. S15k, l), in agreement with the selective effect of afatinib in behaviors. Together, these results suggest a necessary role of VTA ErbB4 kinase activity in CSDS-induced depression-like behaviors.

**Fig. 5:**
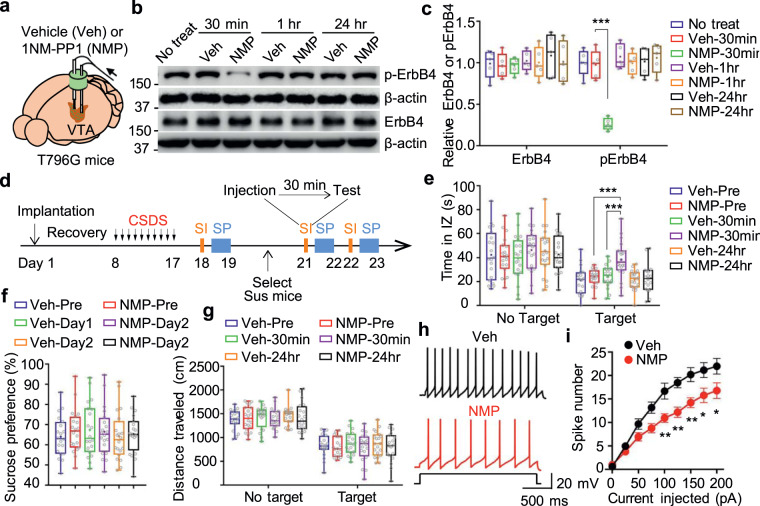
Attenuated expression of depression-like behaviors after acute injection of 1NMPP1 into VTA of CSDS-susceptible mice. **a** Acute injection of NMP or Veh into VTA via implanted cannulae. **b, c** Acute inhibition of pErbB4 in VTA by single injection of NMP. **b** Representative western blots. **c** Quantitative data in **b**. Data were expressed as box-and-whisker plots. One-way ANOVA, *F*_(6, 28)_ = 18.87, ***P < 0.0001, Sidak’s multiple comparisons test, ***P_(Veh-30 min vs. NMP-30 min)_ < 0.0001; *n* = 5 mice for each time point. **d** Time scheme of behavioral studies. SI social interaction test, SP sucrose preference test, Sus susceptible. **e** Attenuation of CSDS-induced social avoidance by single administration of NMP into VTA. No difference was observed for time spent in social target-absent IZ. Data were expressed as box-and-whisker plots. At 30 min, unpaired Student’s *t* test, *t*_38_ = 3.488, **P = 0.0012. For comparison between different time points of NMP group, one-way ANOVA, *F*_(2, 57)_ = 11.03, ***P < 0.0001, ***P_(Pre vs. 30 min)_ = 0.0008. *n* = 20 mice per group. **f** No alteration by single injection of NMP in CSDS-induced reduction of sucrose preference (tested during two consecutive 24-h periods). **g** No alteration in locomotion. **h, i** Inhibition of DA neuron firing in VTA slices prepared from CSDS-susceptible mice by incubation with NMP. **h** Representative traces of current injection-induced spikes. **i** Quantification of **h**. Data were expressed as mean ± SEM. Two-way ANOVA, *F*_(interaction)(8, 280)_ = 1.996, *P = 0.0470; Sidak’s multiple comparisons test, **P_(100 pA)_ = 0.0068, **P_(125 pA_) = 0.0020, **P_(150 pA)_ = 0_._0040, *P_(175 pA)_ = 0.0140, *P_(200 pA)_ = 0.0191; *n* = 19 (Veh) or 18 (NMP) cells from 8 mice. (Also see Supplementary Fig. S15 for attenuation of depression-like behaviors by single administration of afatinib into VTA.).

Although afatinib is able to inhibit ErbB4, it has lower EC50 values for ErbB1 [82], and thus may have off-target effects. To convincingly demonstrate that ErbB4 kinase activity is critical to the expression of CSDS-induced depression-like behaviors, we studied T796G mice. T796G mice were subjected to CSDS, and susceptible mice were injected with 1NMPP1 into bilateral VTAs via cannulae (Fig. 5a). pErbB4 in VTA was reduced by 1NMPP1 (500 nM, 200 nl) within 30 min and recovered afterwards (Fig. 5b, c) [27]. Therefore, social avoidance in CSDS-susceptible mice was tested at 30 min after injection (Fig. 5d). As shown in Fig. 5e and Supplementary Fig. S4d, the time spent in the interaction zone with social target was increased in 1NMPP1-injected T796G mice, compared with Veh-treated mice. 1NMPP1 injection seemed to have little effect on locomotor activity (Fig. 5g). These results suggest a critical role of ErbB4 kinase activity in the expression of depression-like behavior. In agreement, the attenuating effect of 1NMPP1 was not observed 24 h after injection when ErbB4 kinase activity was already recovered (Fig. 5e). Note that sucrose preference was similar between 1NMPP1-injected and Veh-injected T796G mice (Fig. 5f), perhaps because the inhibition of ErbB4 by 1NMPP1 was transient and sucrose preference was a sum of 24 h. We have also investigated the effect of 1NMPP1 on VTA slices from CSDS-susceptible mice. As shown in Fig. 5h, i, the AP spikes in response to injected currents of VTA DA neurons were reduced by 1NMPP1. Together, these results demonstrate a critical role of VTA ErbB4 kinase activity in the expression of CSDS-induced social avoidance.

## Discussion

Our study provides evidence that the NRG1-ErbB4 signaling in the LDTg-VTA circuit is necessary for social stress-induced depression-like behaviors. First, pErbB4 was increased in VTA in susceptible mice after CSDS. Accordingly, DA neuron- or VTA-specific deletion of ErbB4 caused a pro-resilient effect in the development of depression-like behaviors. Similar effects were observed by VTA-specific inhibition of ErbB4 kinase activity. These results suggest that the development of depression-like behaviors requires ErbB4 and its activity in VTA DA neurons. Second, CSDS increased the expression of NRG1 at LDTg; deleting NRG1 in LDTg reduced ErBB4 activation in VTA and CSDS-induced depression-like behaviors. Moreover, virus-mediated expression of NRG1 in LDTg enhanced subSD-induced depression behaviors, along with increased NRG1 protein in VTA. These results identify a novel function of the LDTg neurons that project to VTA DA neurons. Third, mechanistically, NRG1 suppressed K^+^ currents and thus increased the activity of VTA DA neurons, whereas inhibiting ErbB4 increased K^+^ currents and reduced DA neuron firing. Fourth, in accord, acute inhibition of ErbB4 in VTA attenuated the depression-like behaviors in CSDS-susceptible mice. These results demonstrate that the NRG1-ErbB4 signaling at the LDTg-VTA circuit promotes the response of VTA DA neurons to social stress and increases the susceptibility to depression-like phenotypes, revealing a novel mechanism that regulates the development of depression.

Abnormality of VTA DA neuron activity has been implicated in depression. The firing of DA neurons in the VTA is increased in rodents after CSDS and chronic restraint [7, 85, 86], and reduced in chronic mild stress model [8, 87]. Changes in DA neuron activity alter the development and expression of depression-like behaviors [5, 8]. The activity of VTA DA neurons is regulated by inputs from different brain regions. PV neurons of the VP region are increased in activity in susceptible mice after CSDS; optogenetic or chemogenetic inhibition of these PV neurons reduces CSDS-induced social avoidance [12]. On the other hand, the activity of NE neurons in the LC region is increased in resilient mice after CSDS; NE antagonism or depletion blocks the resilience [13, 14]. The burst firing of VTA DA neurons is implicated in encoding rewarding and aversive responses and regulated by neuronal activity in the LDTg [88]. Optogenetic activation of LDTg neurons projecting to VTA promotes reward-related behaviors [89–91]. Activity of LDTg neurons is increased by inescapable footshock, predator odorant, and CSDS [88, 92, 93]. In particular, chemogenetic inhibition or activation of LDTg cholinergic neurons suppresses or enhances, respectively, depression-like behaviors and associated DA neuron activity in the VTA [77]. We showed a novel mechanism for LDTg neurons to regulate the activity of VTA DA neurons. NRG1 protein, as well as pErbB4, was increased in the VTA in mice susceptible to CSDS (Figs. 3a, b and 1d, e). However, NRG1 mRNA was not increased in the VTA (Fig. 3b), but in LDTg and DRN regions (Fig. 3d). Specific ablation of the NRG1 gene in LDTg, but not DRN, reduced NRG1 protein and pErbB4 in VTA (Fig. 3e–m) and inhibited depression-like behaviors (Fig. 3n–q). On the other hand, overexpressing NRG1 in LDTg increased NRG1 protein in the VTA (Supplementary Fig. S12e, f) and enhanced depression-like behaviors after subSD induction (Fig. 3r–y). These observations suggest that in response to stress, NRG1 is utilized as a trophic factor for LDTg neurons to regulate the activity of VTA DA neurons. This notion is consistent with earlier findings that the transcription of the NRG1 gene and expression of its protein are dependent on neuronal activity [71–73].

K^+^ currents in VTA DA neurons are increased in resilient mice, which results in an attenuated activity of DA neurons and reduced depression-like behaviors in response to social stress [7, 9, 64]. In susceptible mice, DA neurons seemed to have a problem with increasing K^+^ currents [9]; increasing K^+^ currents and subsequent reduction in DA neuron activity in susceptible mice were shown to cause a pro-resilient effect [7, 64]. Nevertheless, regulatory mechanisms of K^+^ currents of VTA DA neurons in response to stress are not understood. Interestingly, NRG1 in LDTg and pErbB4 in VTA were increased in susceptible mice (Figs. 1 and 3). NRG1 and ErbB4 inhibition were able to suppress and increase, respectively, K^+^ currents in VTA DA neurons (Figs. 2o–q and 4h–j; Supplementary Figs. S9 and S14) and associated changes in neuronal excitability (increased by NRG1 and decreased by ErbB4 inhibition) (Figs. 2i–n and 4a–g). Notably, perturbing the NRG1-ErbB4 signaling attenuated the development of depression-like behaviors (Figs. 1–3, 5 and Supplementary Fig. S15). ERK activity in the VTA has been implicated in regulating responses to social stress [80, 81]; ERK blockade reduces the firing frequency of VTA DA neurons [80, 94] and increases *I*_*A*_ K^+^ currents [95, 96]. We showed that the administration of NRG1 or 1NMPP1 into the VTA increased or decreased, respectively, ERK phosphorylation (Fig. 4k–m); pharmacological inhibition of ERK reduced the effect of NRG1 on DA neuron firing and K^+^ currents (Fig. 4n–q). A parsimonious interpretation of these results is that the NRG1-ErbB4 signaling promotes VTA DA neuron activity via an ERK-dependent inhibition of K^+^ channels.

In short, we demonstrate a role of the NRG1-ErbB4 signaling in the development and expression of depression-like behaviors in response to social stress, and reveal an unexpected mechanism by which the LDTg-VTA circuit regulates VTA DA neuron activity. *NRG1* and *ERBB4* were identified as risk genes for schizophrenia [15, 18, 97–99], although the association was not observed by a recent GWAS of large samples [100]. However, both *NRG1* and *ERBB4* were among the 269 putative genes identified by GWAS of 246,363 depression patients [36]. Responses to classical antidepressants were predicted by GWAS-identified genetic variants of *NRG1* and *ERBB4* [37–39]. Furthermore, ketamine, an antidepressant, was found to alter the levels of NRG1 or ErbB4 in the cortex and hippocampus [101–103]; exogenous NRG1 blocked the reactivation effect of ketamine on cortical plasticity [102], a potential mechanism for the antidepressant effect of ketamine [104]. Therefore, our studies provide insight into pathological mechanisms of relevant types of depression.

## Supplementary information

Supplementary Figures and legends
