# Supplementary material for: The laterodorsal tegmentum-ventral tegmental area circuit controls depression-like behaviors by activating ErbB4 in DA neurons
Source: Mol Psychiatry. 2021 May 14;28(3):1027–45. doi: 10.1038/s41380-021-01137-7 (PMC8590712; doi:10.1038/s41380-021-01137-7)

Supplementary Table 1. Changes in passive and active membrane properties by NRG1 and 1NMPP1. RMP, resting membrane potential; Ri, input resistance; Cm, membrane capacitance; AHP, after-hyperpolarization. AP width was measured at the level of AP threshold. First spike latency is the time between current injection and appearance of the first spike. Data were presented as Mean ± SEM, n = 19 - 20, Student's t-test. $t_{AHP(baseline\ vs.\ NRG1)(36)} = 2.178$, *P = 0.0361; $t_{AHP(baseline\ vs.\ 1NMPP1)(38)} = 2.033$, *P = 0.0491; $t_{latency(baseline\ vs.\ NRG1)(36)} = 2.293$, *P = 0.0278; $t_{latency(baseline\ vs.\ 1NMPP1)(38)} = 2.842$, **P = 0.0072.

Supplementary Fig. S1. Segregation of mice that were susceptible and resilient to the CSDS. Mice were segregated into different subpopulations based on the social avoidance test after CSDS, followed by sucrose consumption test and western blot analysis.

Supplementary Fig. S2. Selective targeting of DA neurons in DAT-Cre mice. **a** DAT-Cre mice crossed with Ai9 reporter mice. **b** Images showing overlap between tdTomato+ (referred to as tdT) cells and TH+ cells, scale bars, upper: 200 μm, lower: 50 μm; **c** Quantitative data of b.

Supplementary Fig. S3. No alteration in TH expression in VTA in the CKO mice. **a** Representative western blot bands. **b** Quantification of a.

Supplementary Fig. S4. Tracking images of the social avoidance tests. **a** CKO mice, related to Fig. 1k; **b** mice of LDTg-specific ablation of NRG1, related to Fig. 3o; **c** mice of LDTg-specific overexpression of NRG1, related to Fig. 3t; **d** CSDS-susceptible mice that received VTA-specific injection of NMP, related to Fig. 5e.

Supplementary Fig. S5. No alteration in locomotion in the CKO mice. Quantification of traveled distance during the social avoidance test showing no difference between

CKO and control (Cre) mice. CKO, conditional knockout (DAT-Cre+/-;ErbB4f/f) mice; Cre, DAT-Cre+/- mice.

Supplementary Fig. S6. Expression of AAV in VTA. **a** Anatomic diagram showing location of VTA in brain slices from rostral to caudal. **b** Low power images present in rostral to caudal order showing expression of AAV-GFP at VTA; Scale bar, 1 mm. **c-d** Amplification of the rectangular area in b; Scale bars, 100 μm in c, 50 μm in d. **e** Low power images showing expression of AAV-Cre at VTA; Scale bar, 1 mm. **f-g** Amplification of rectangular area in e; Scale bars, 100 μm in f, 50 μm in g.

Supplementary Fig. S7. Verification of positions of cannulae implanted into VTA. **a** Corresponding to Fig. 3, osmotic pump-driven continuous infusion of vehicle (Veh) or 1NM-PP1 (NMP) to VTA; numbers on the left indicate anterior-posterior (AP) coordinates, unit: mm. **b** Corresponding to Fig. 7, injection of Veh, NRG1, or NMP into VTA followed by detection of ERK and pERK. **c** Corresponding to Fig. 8, acute injection of Veh or NMP into VTA of CSDS-susceptible (Sus) mice, followed by test of depression-like behaviors. **d** Corresponding to supplementary Fig. S4, injection of Veh, NRG1, or NMP into VTA followed by detection of BDNF at NAc, with the left and right hemisphere for Western blot and qPCR analysis, respectively. **e** Corresponding to supplementary Fig. S8, acute infusion of Veh, afatinib (Afa) or lapatinib (Lap) to VTA of CSDS-susceptible (Sus) mice, followed by test of depression-like behaviors.

Supplementary Fig. S8. No changes in synaptic inputs onto VTA DA neurons after incubation of NRG1 and 1NMPP1. DAT::Cre;Ai9 mice were crossed to T796G mice and recording was performed in tdTomato-labeled cells. **a** No changes in sEPSC frequency and amplitude after incubation of NRG1 or 1NMPP1; upper, representative traces, lower, quantification data. **b** No changes in mEPSC frequency

and amplitude after incubation of NRG1 or 1NMPP1; upper, representative traces, lower, quantification data. **c** No changes in sIPSC frequency and amplitude after incubation of NRG1 or 1NMPP1; upper, representative traces, lower, quantification data. **d** No changes in mIPSC frequency and amplitude after incubation of NRG1 or 1NMPP1; upper, representative traces, lower, quantification data.

Supplementary Fig. S9. Enhancement of I_A activation by 1NMPP1 in VTA DA neurons. DAT::Cre;Ai9 mice were crossed to T796G mice and recording was performed in tdTomato-labeled cells. **a** Representative traces of I_A activated by series of depolarizing voltage steps after incubation with Vehicle (left) or 1NMPP1 (right). **b** Right shift of the steady-state activation curve of I_A by incubation with 1NMPP1; V_h and k value were obtained for each recorded cell and Student's t-test was performed to analyze the difference between groups; for V_h, $t_{(Vehicle\ vs.\ 1NMPP1)(16)} = 3.01$, *P = 0.008. **c** No effect of 1NMPP1 on steady-state inactivation curve of I_A. **d** Representative traces of I_{DR} activated by series of depolarizing voltage steps after incubation with Vehicle (left) or 1NMPP1 (right). **e** No effect of 1NMPP1 on steady-state activation curve of I_{DR}.

Supplementary Fig. S10. Promotion and inhibition of BDNF release at NAc by VTA-injected NRG1 and 1NM-PP1, respectively. **a-b** subSD- or CSDS-exposed mice received consecutive infusion of NRG1 (10nM) or 1NM-PP1 (NMP) (100 nM) into VTA, followed by dissection of NAc 24 hrs later [64]. **c-d** Increased BDNF protein in NAc of subSD-exposed mice by NRG1, while decreased BDNF protein in NAc of CSDS-exposed mice by NMP. **c** representative Western blots for dissected NAc. **d** quantitative data in c. Data were expressed as box-and-whisker plots. One-way ANOVA, $F_{(4, 43)} = 6.83$, ***P = 0.0002; $P_{(subSD+Veh\ vs.\ Naive)} = 0.9997$, *$P_{(subSD+NRG1\ vs.\ subSD+Veh)} = 0.0360$, **$P_{(CSDS+Veh\ vs.\ Naive)} = 0.0016$, **$P_{(CSDS+Veh\ vs.\ subSD+Veh)} = 0.0003$,

$P_{(\text{CSDS+NMP vs. CSDS+Veh})}$ = 0.0063. n = 8 (naive) or 10 mice per group. **e No alteration in BDNF mRNA in NAc, n = 8 mice per group.

Supplementary Fig. S11. AAV expression at LDTg and DRN. **a** Anatomic diagram showing location of LDTg in brain slices from rostral to caudal. **b** Representative images present in rostral to caudal order showing AAV-GFP expression at LDTg; scale bar, 1 mm. **c** Anatomic diagram showing location of DRN in brain slices from rostral to caudal. **d** Representative images present in rostral to caudal order showing expression of AAV-GFP at DRN. Scale bar, 1 mm.

Supplementary Fig. S12. Changes in total ErbB4 protein and NRG1 protein in VTA of CSDS or subSD-exposed mice after deletion or overexpression of NRG1 in LDTg. **a** Representative Western blots for dissected VTA after AAV-Cre (vCre) injection into LDTg of NRG1f/f mice. Mice after indicated paradigms were analyzed. **b** Quantification data in A. **c** Representative Western blots for dissected VTA after vCre injection into DRN of NRG1f/f mice. Mice after indicated paradigms were analyzed. **d** Quantification data in c. n.s., no significant difference. **e** Representative Western blots of dissected VTA after injection of AAV-GFP (vGFP) or AAV-NRG1-GFP (vNRG1) into LDTg, without or with subSD exposure. **f** Quantification of e. Increase of NRG1 in VTA was observed in exposing vNRG1-injected mice to subSD, while vGFP-injected subSD mice or vNRG1-injected naive mice showed no increase of NRG1 in VTA. Data were expressed as box-and-whisker plots. One-way ANOVA, $F_{(3, 24)}$ = 3.429, *P = 0.0331; Sidak's multiple comparisons test, *$P_{(\text{Naive-vGFP vs. subSD-vNRG1})}$ = 0.0350; n = 7 mice per group.

Supplementary Fig. S13. Genetic labeling of ErbB4-expressing DA neurons in VTA. **a** ErbB4-CreER mice crossed to Ai9 reporter mice to generate the ErbB4-tdTomato mice. **b** Time scheme for tamoxifen injection and collection of samples. **c** Region of

VTA that was imaged. **d** Representative images showing localization of tdTomato (tdT) signals in tyrosine hydroxylase (TH)-labeled DA neurons in VTA. Scale bars, 100 μm for upper, 25 μm for lower. **e** Quantification of the tdT-labeled TH+ neurons.

Supplementary Fig. S14. Inhibition of activation of I_A by NRG1 in VTA DA neurons. TdTomato+ cells in VTA slices prepared from ErbB4::CreER;Ai9 mice were recorded and back labeled with biocytin and stained with anti-TH antibody. Results from tdTomato and TH double positive cells were used for analysis. **a** Representative traces of I_A activated by series of depolarizing voltage steps after incubation with Vehicle (left) or NRG1 (right). **b** Right shift of the steady-state activation curve of I_A by incubation with NRG1; V_h and k value were obtained for each recorded cell and Student's t-test was performed to analyze the difference between groups; for V_h, $t_{(\text{Vehicle vs. NRG1})(18)} = 2.286$, *P = 0.0346. **c** No effect of NRG1 on steady-state inactivation curve of I_A. **d** Representative traces of I_{DR} activated by series of depolarizing voltage steps after incubation with Vehicle (left) or NRG1 (right). **e** No effect of NRG1 on steady-state activation curve of I_{DR}.

Supplementary Fig. S15. Reduced expression of depression-like behaviors after acute injection of afatinib into VTA of CSDS-susceptible mice. **a** Diagram showing acute injection of afatinib (Afa), Lapatinib (Lap), or vehicle (Veh) into VTA via implanted cannulae. **b-c** Rapid and endured suppression of pErbB4 in VTA by single administration of Afa, but not Lap, into VTA. **b** representative Western blots for dissected VTA. **c** quantification of pErbB4. Data were expressed as box-and-whisker plots. One-way ANOVA, $F_{(10, 44)} = 74.27$, ****P < 0.0001; Sidak's multiple comparisons test, ***$P_{(\text{Afa vs. Veh})} < 0.001$. **d** Time scheme for the behavioral studies; SI, social interaction test; SP, sucrose preference test; Sus, susceptible. **e** No alteration in time spent in social target-absent IZ after drug administration. Pre,

pretreatment. **f** Attenuation of CSDS-induced social avoidance after single injection of Afa, not Lap, into VTA. Data were expressed as box-and-whisker plots. At 30 min, one-way ANOVA, $F_{(2, 57)} = 12.51$, ***$P < 0.0001$, Sidak's multiple comparisons test, ***$P_{(Veh\ vs.\ Afa)} = 0.0003$, $P_{(Veh\ vs.\ Lap)} = 0.9935$, ***$P_{(Afa\ vs.\ Lap)} = 0.0001$. At 24 hr, one-way ANOVA, $F_{(2, 57)} = 7.571$, **$P = 0.0012$, Sidak's multiple comparisons test, **$P_{(Veh\ vs.\ Afa)} = 0.0065$, $P_{(Veh\ vs.\ Lap)} = 0.9877$, **$P_{(Afa\ vs.\ Lap)} = 0.0027$. Comparison between different time points of Afa group, one-way ANOVA, $F_{(3, 76)} = 6.288$, $^{###}P = 0.0007$, Sidak's multiple comparisons test, $^{##}P_{(Pre\ vs.\ 30\ min)} = 0.0021$, $^{#}P_{(Pre\ vs.\ 24\ hr)} = 0.0252$. n = 20 mice per group. **g-h** No alteration in locomotion after single injection of Afa or Lap into VTA. **i** Attenuation of CSDS-induced reduction of sucrose preference by single administration of Afa, not Lap, into VTA. Data were expressed as box-and-whisker plots. One-way ANOVA, for day 1, $F_{(2, 57)} = 4.988$, *$P = 0.0101$, Sidak's multiple comparisons test, *$P_{(Veh\ vs.\ Afa)} = 0.0400$, *$P_{(Afa\ vs.\ Lap)} = 0.0163$; for comparison between different time points of Afa group, $F_{(2, 57)} = 3.64$, $^{#}P = 0.0325$, Sidak's multiple comparisons test, $^{#}P_{(Pre\ vs.\ Day\ 1)} = 0.0282$. n = 20 mice per group. **j-l** Suppression of DA neuron firing in VTA slices prepared from CSDS-susceptible mice by incubation with Afa, not Lap. **j** diagram showing recording in VTA slices. **k** representative traces of current injection-induced spikes. **l** quantification of k. Data were expressed as mean ± SEM. Two-way ANOVA; Veh vs. Afa, $F_{(interaction)(8, 288)} = 2.411$, *$P = 0.0156$; Sidak's multiple comparisons test, **$P_{(100\ pA)} = 0.0033$, ***$P_{(125\ pA)} = 0.0002$, ***$P_{(150pA)} = 0.0001$, **$P_{(175\ pA)} = 0.0016$, **$P_{(200pA)} = 0.0012$; Afa vs. Lap, , $F_{(interaction)(8, 272)} = 1.982$, $^{#}P = 0.0488$; Sidak's multiple comparisons test, $^{##}P_{(75\ pA)} = 0.0072$, $^{##}P_{(100\ pA)} = 0.0022$, $^{##}P_{(125\ pA)} = 0.0013$, $^{##}P_{(150\ pA)} = 0.0045$; n = 20 (Veh) or 18 (Afa, Lap) cells from 8 mice.

Supplementary Table 1

	RMP (mV)	Ri (MΩ)	Cm (pF)	AP Threshold (mV)	AP Amplitude (mV)	AP Duration (ms)	AHP (mV)	First spike latency (ms)
Baseline	56.6 ± 1.37	432 ± 14.5	49.7 ± 1.63	-30.4 ± 0.79	65.6 ± 1.27	2.64 ± 0.15	-62.6 ± 1.47	148 ± 10.4
NRG1	54.6 ± 1.08	411 ± 10.7	38.6 ± 1.77	-31.2 ± 0.73	64.4 ± 1.41	2.52 ± 0.16	-58.5 ± 1.14*	111 ± 12.2*
Baseline	55.1 ± 1.68	419 ± 13.5	37.0 ± 1.92	-32.3 ± 0.80	62.3 ± 1.29	2.47 ± 0.15	-62.7 ± 1.08	152 ± 7.86
1NMPP1	58.3 ± 1.25	445 ± 11.1	39.1 ± 1.70	-31.0 ± 0.76	64.7 ± 1.62	2.40 ± 0.17	-66.1 ± 1.33*	185 ± 9.79**

Supplementary Fig. S1

a

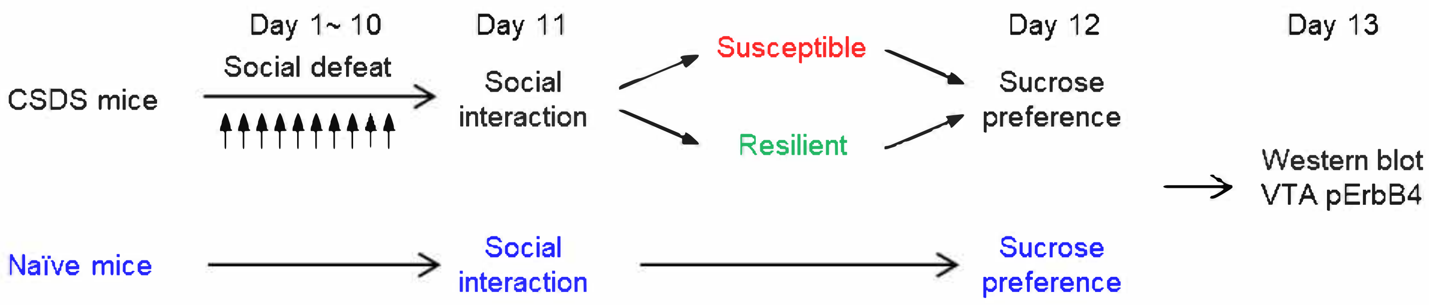

Supplementary Fig. S2

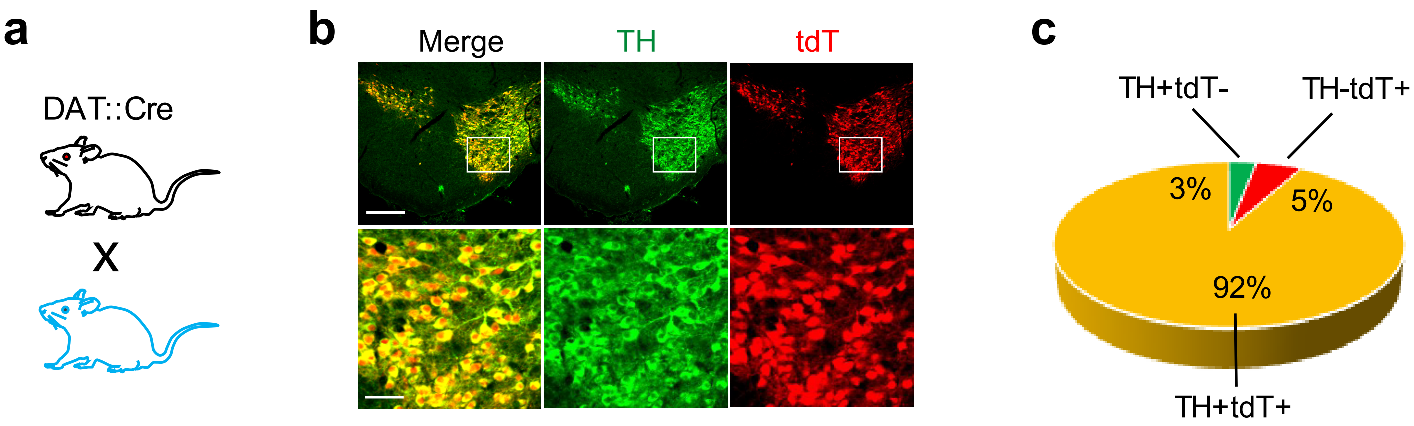

Supplementary Fig. S3

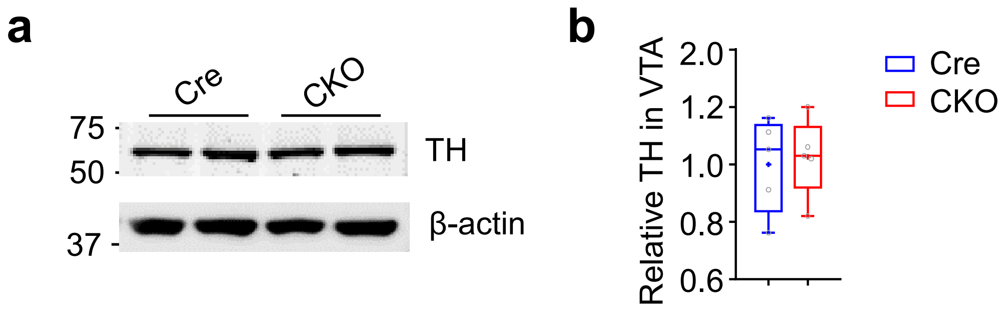

Supplementary Fig. S4

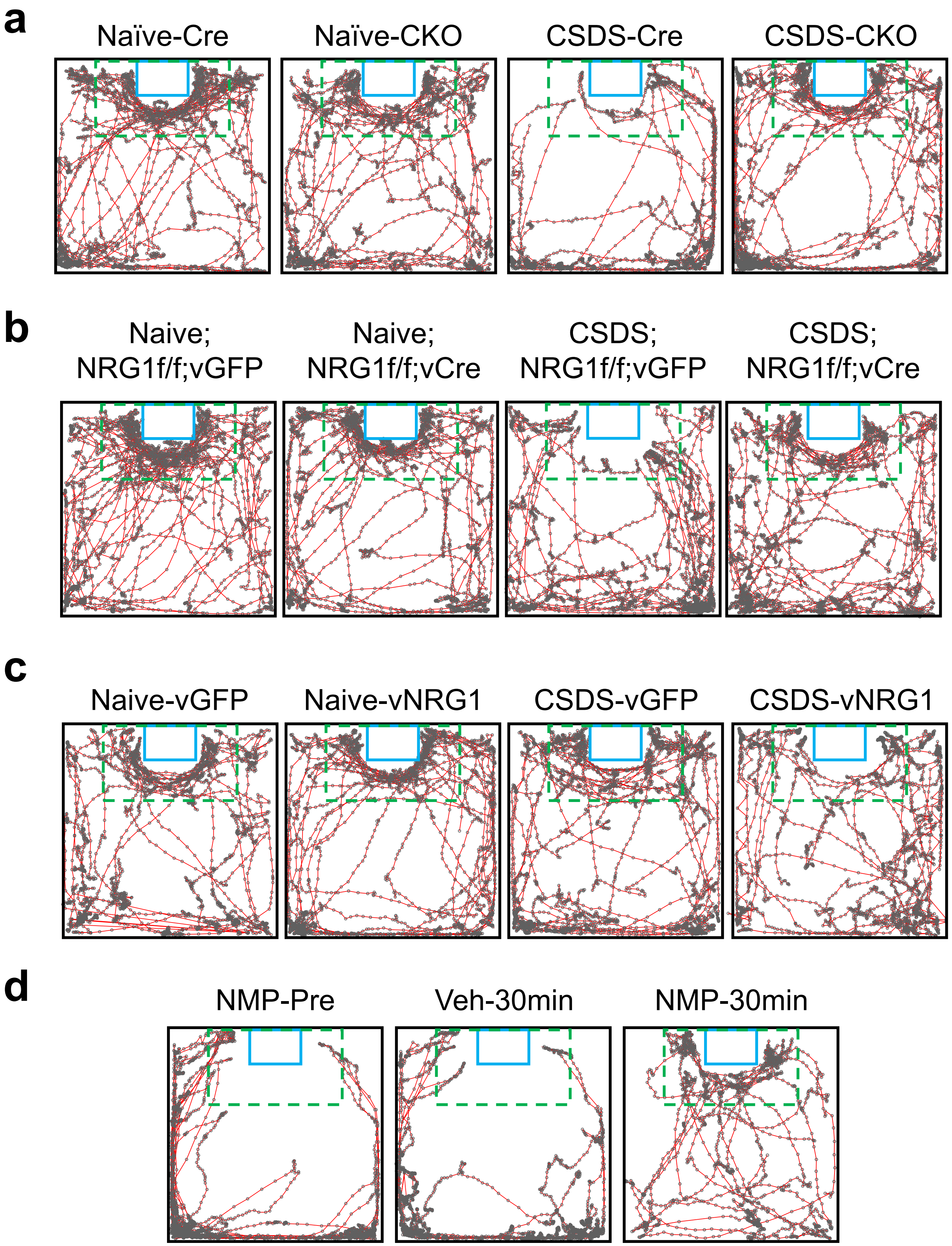

Supplementary Fig. S5

a

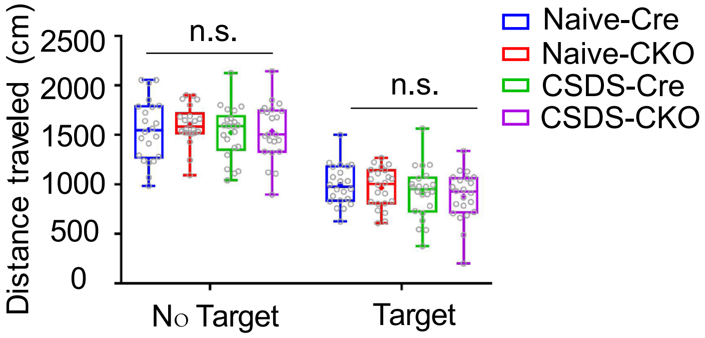

Supplementary Fig. S6

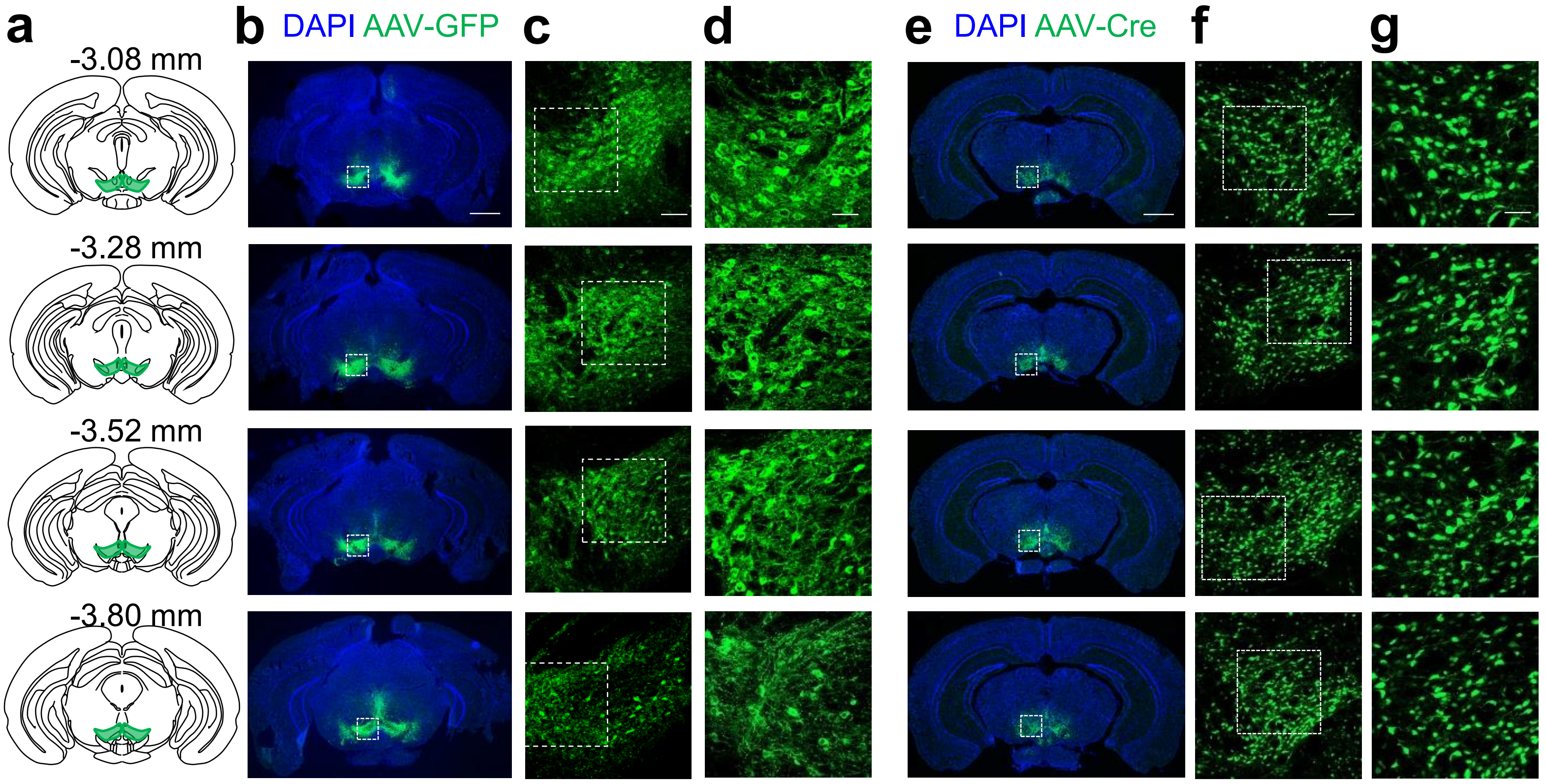

Supplementary Fig. S7

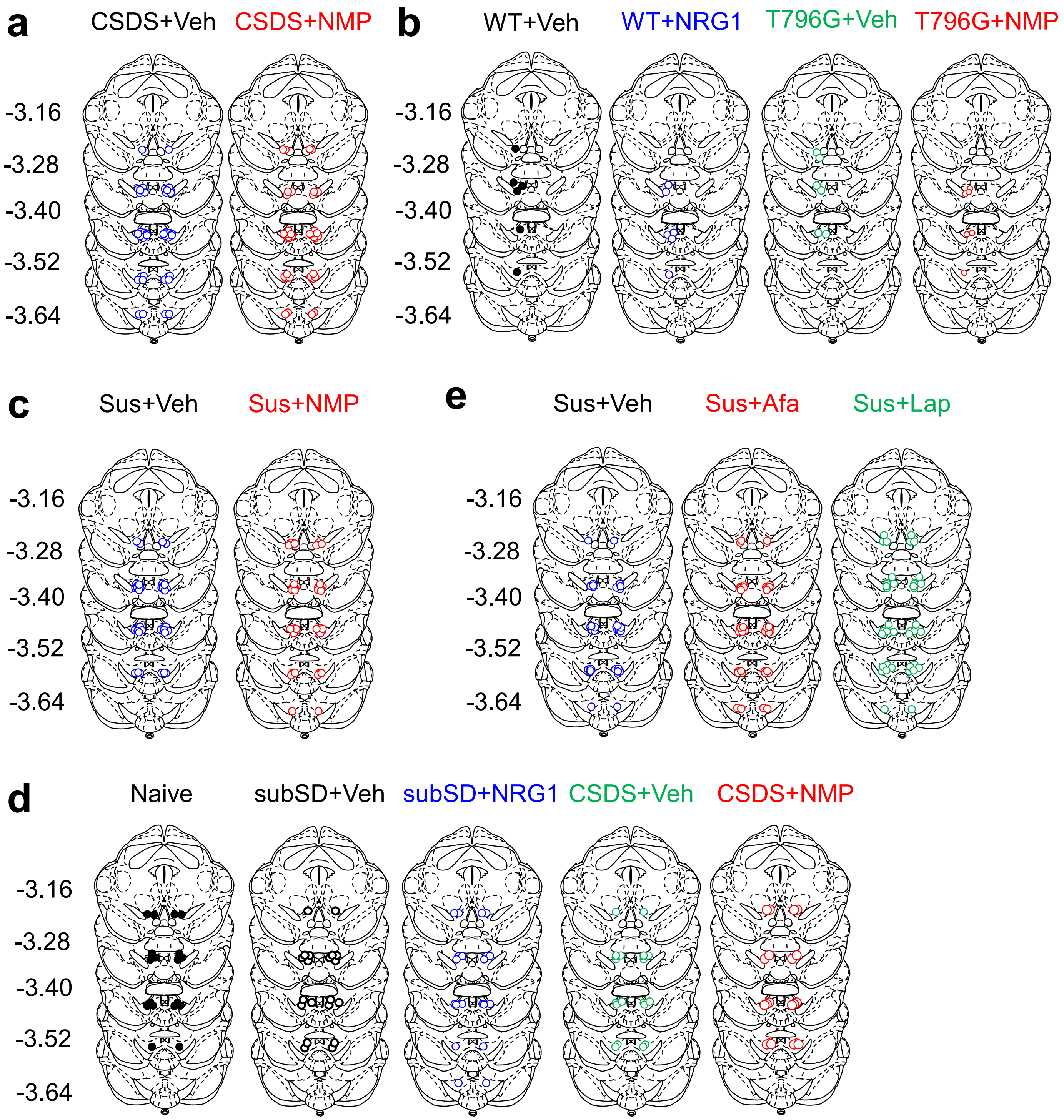

Supplementary Fig. S8

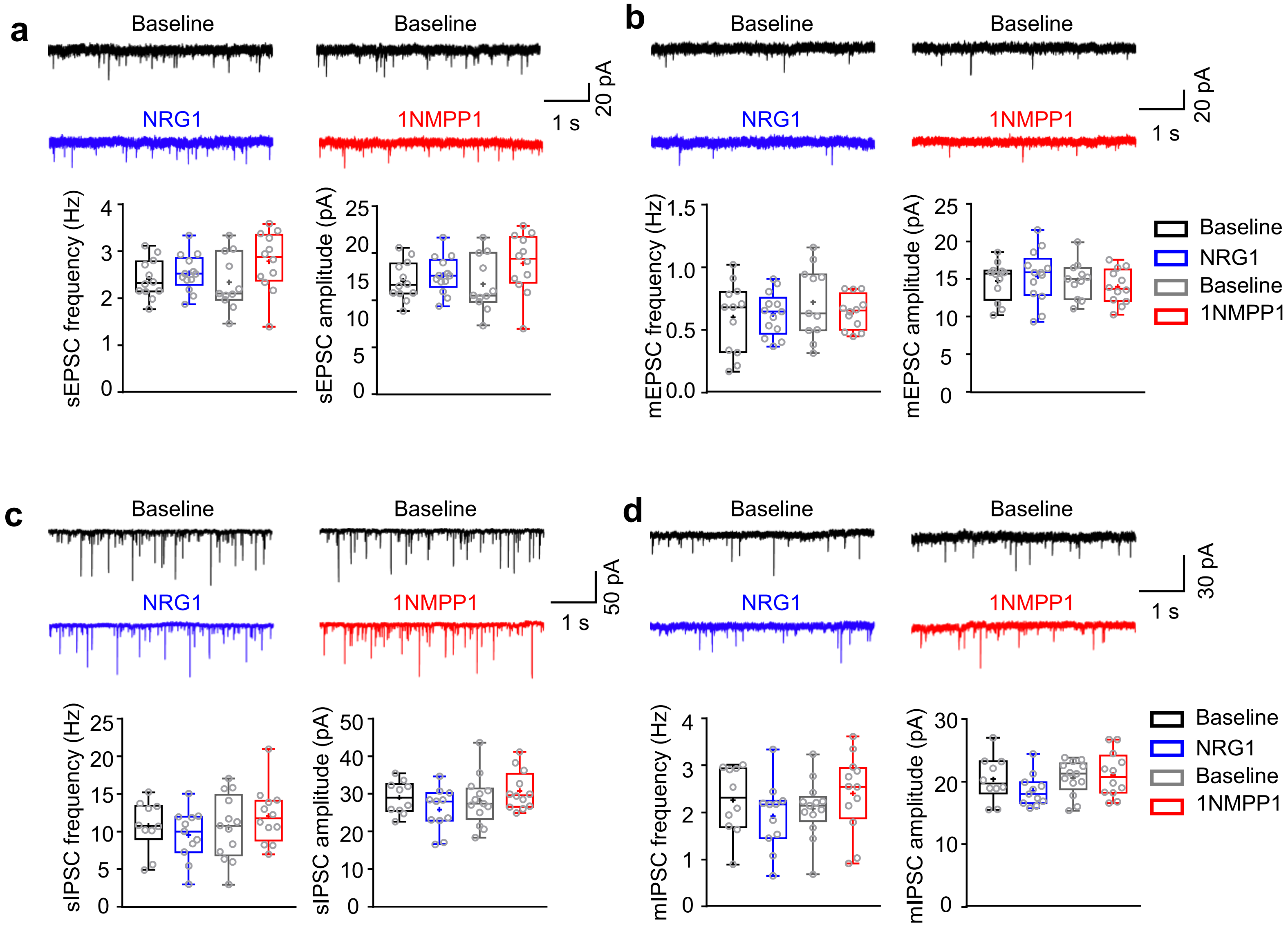

Supplementary Fig. S9

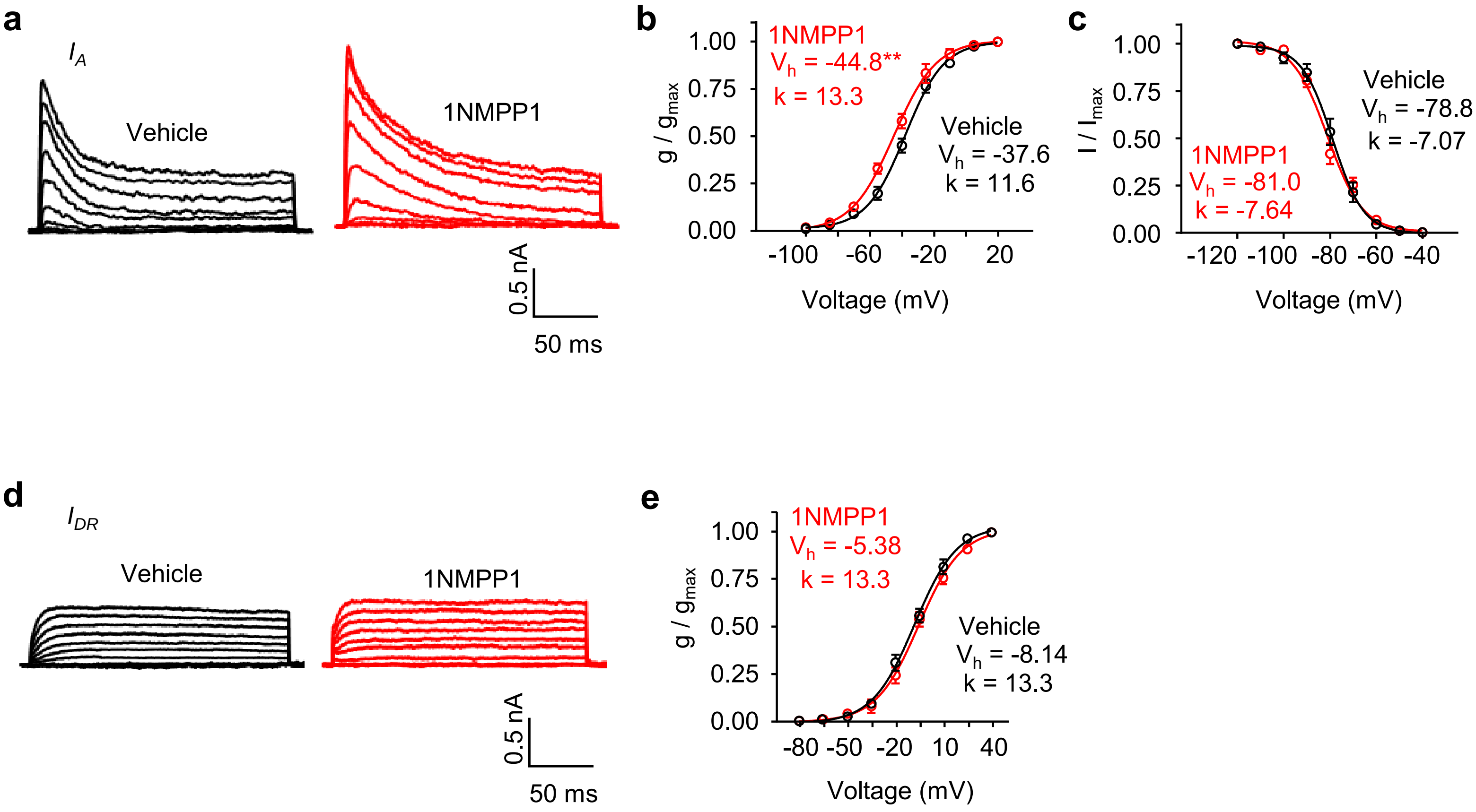

Supplementary Fig. S10

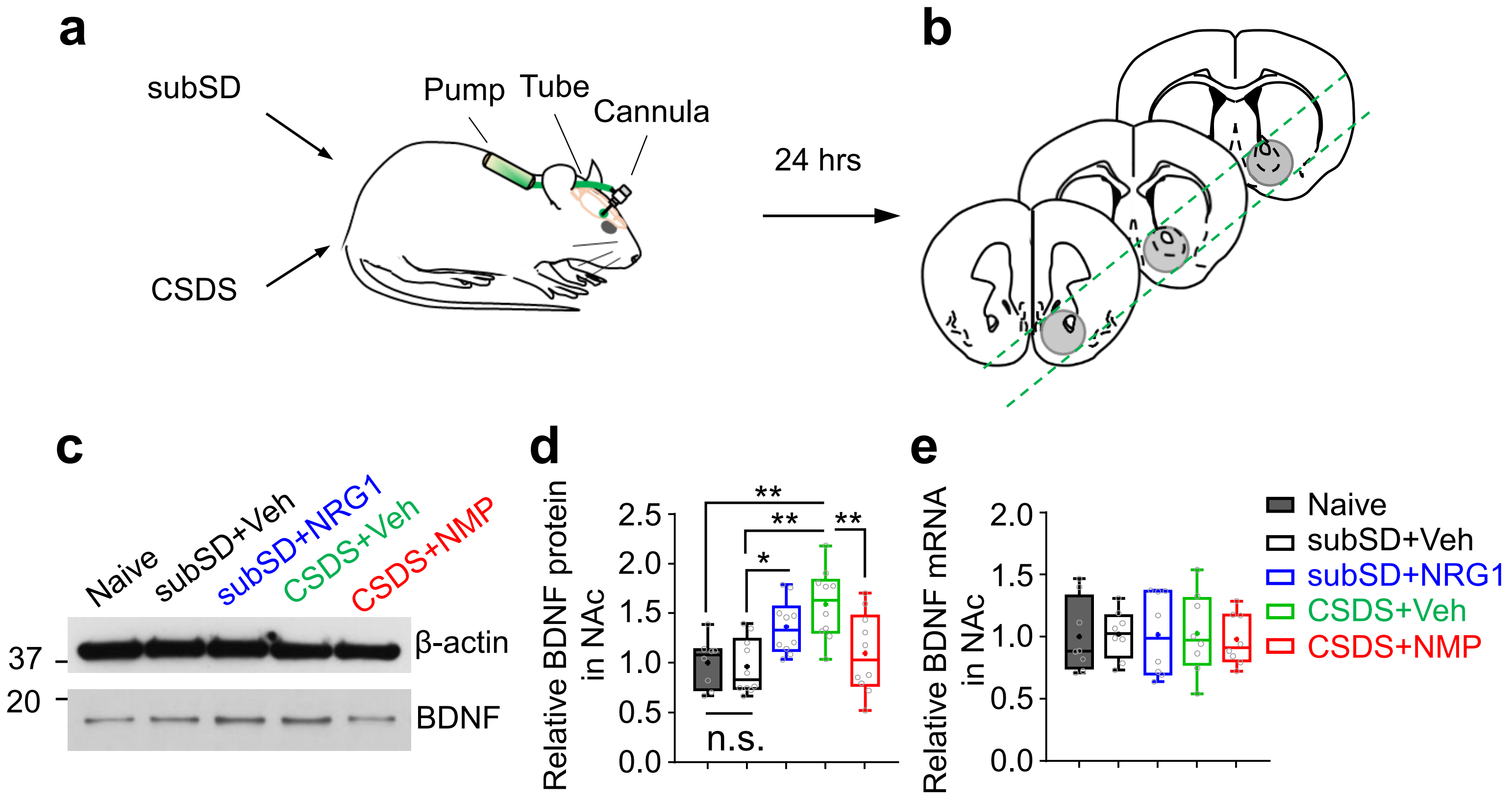

Supplementary Fig. S11

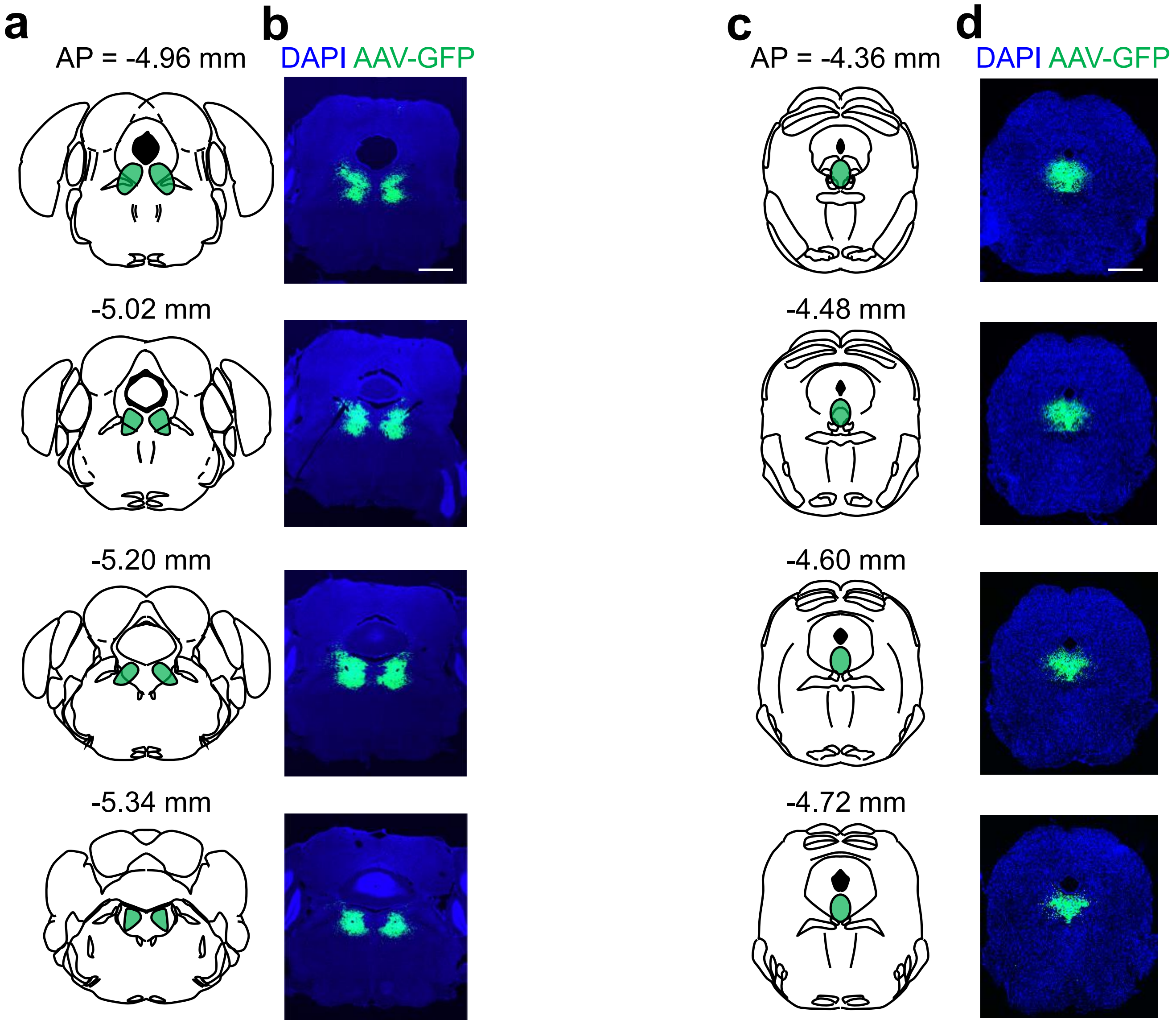

Supplementary Fig. S12

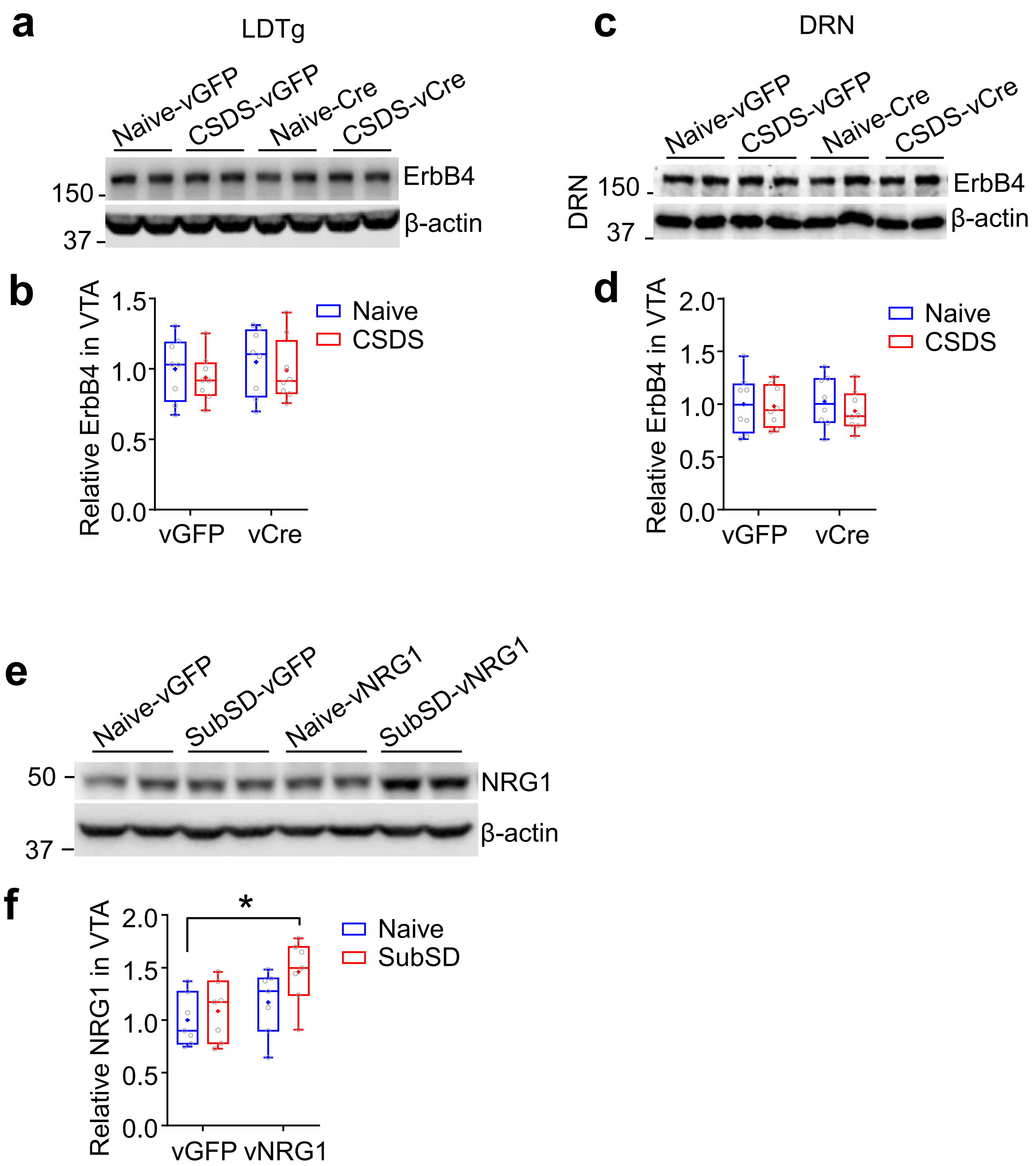

Supplementary Fig. S13

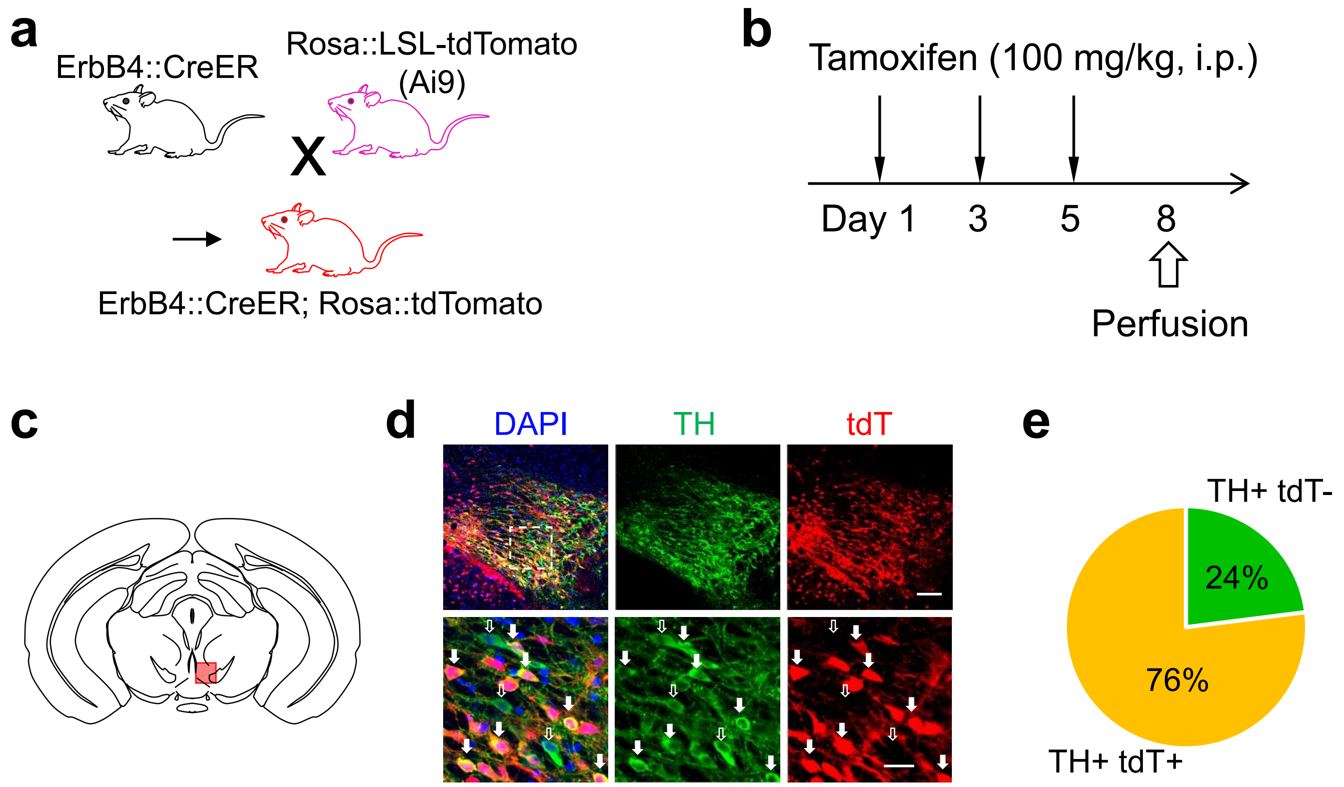

Supplementary Fig. S14

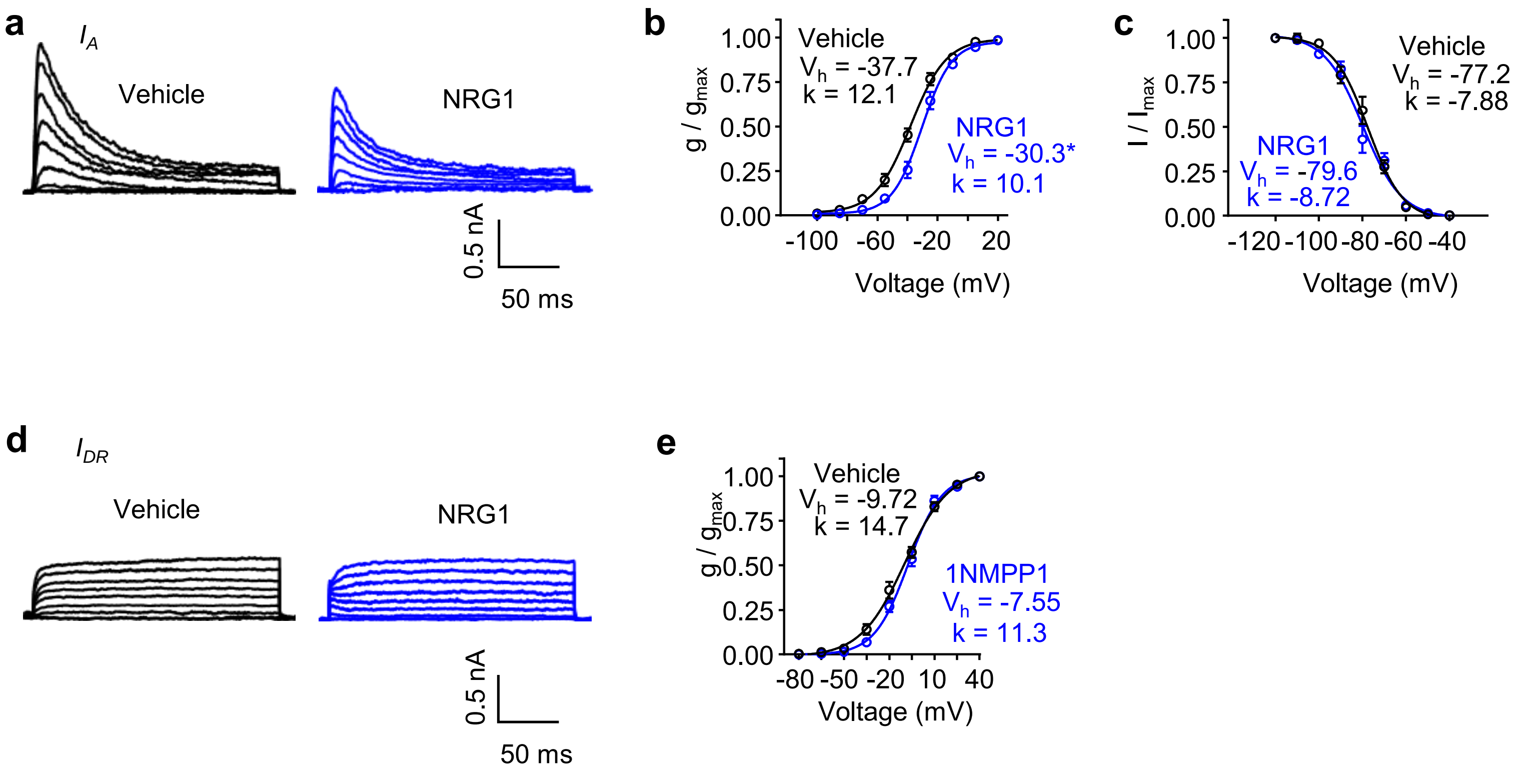

Supplementary Fig. S15

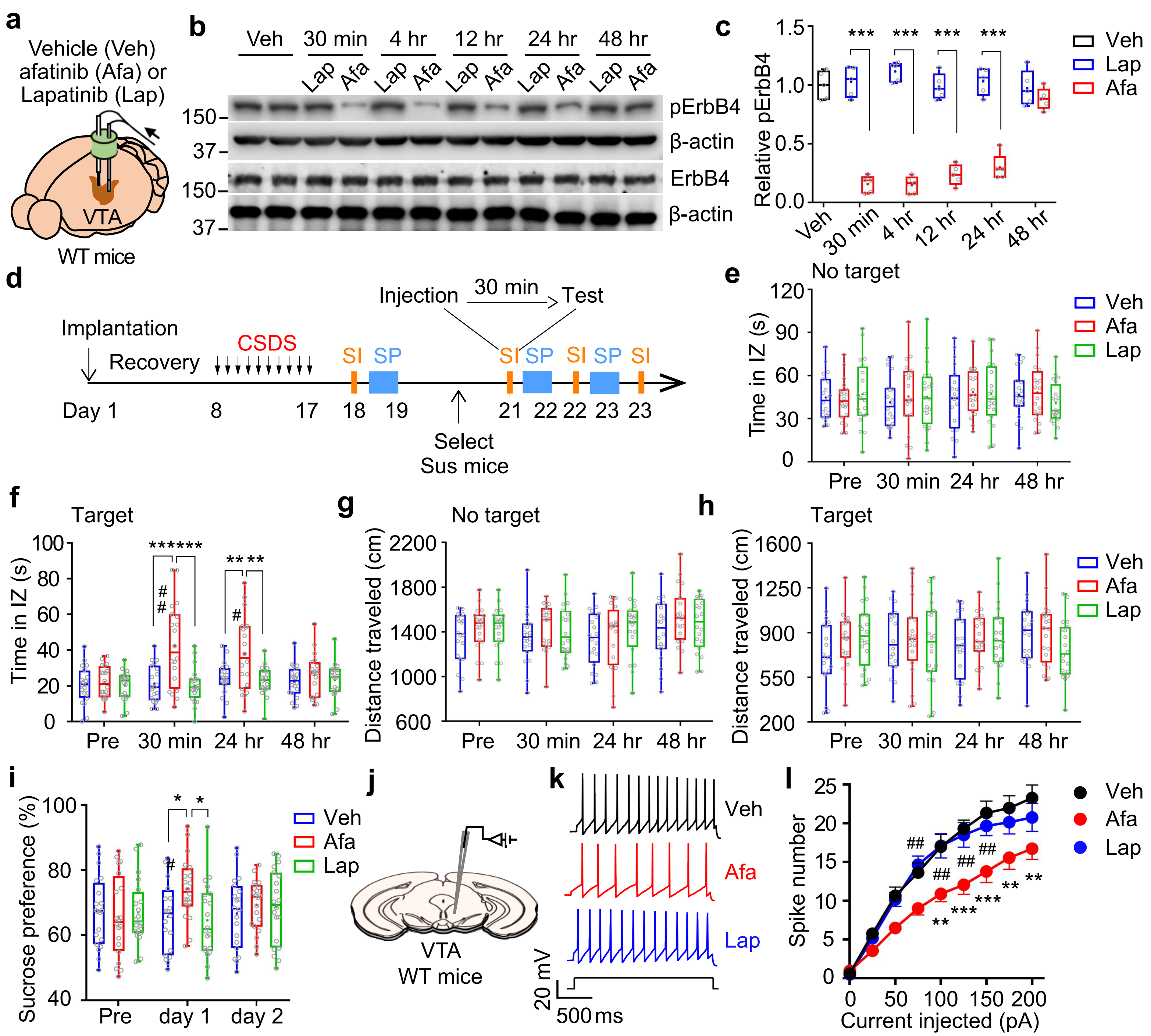

Supplement: Supplementary file 1 — Supplementary Figures and legends [file 41380_2021_1137_MOESM1_ESM.pdf]
